# ROS-responsive 18β-glycyrrhetic acid-conjugated polymeric nanoparticles mediate neuroprotection in ischemic stroke through HMGB1 inhibition and microglia polarization regulation

**DOI:** 10.1016/j.bioactmat.2022.03.040

**Published:** 2022-04-01

**Authors:** Lulu Jin, Zhixin Zhu, Liangjie Hong, Zhefeng Qian, Fang Wang, Zhengwei Mao

**Affiliations:** a MOE Key Laboratory of Macromolecular Synthesis and Functionalization, Department of Polymer Science and Engineering, Zhejiang University, Hangzhou, 310027, China; b The MOE Frontier Science Center for Brain Research and Brain-Machine Integration, Zhejiang University School of Brain Science and Brain Medicine, Hangzhou, 310058, China

**Keywords:** HMGB1, Microglia, M1/M2 phenotype, Polymer-drug conjugates, Drug delivery, Ischemic stroke

## Abstract

Ischemic stroke is an acute and serious cerebral vascular disease, which greatly affects people's health and brings huge economic burden to society. Microglia, as important innate immune components in central nervous system (CNS), are double-edged swords in the battle of nerve injury, considering their polarization between pro-inflammatory M1 or anti-inflammatory M2 phenotypes. High mobility group box 1 (HMGB1) is one of the potent pro-inflammatory mediators that promotes the M1 polarization of microglia. 18β-glycyrrhetinic acid (GA) is an effective intracellular inhibitor of HMGB1, but of poor water solubility and dose-dependent toxicity. To overcome the shortcomings of GA delivery and to improve the efficacy of cerebral ischemia therapy, herein, we designed reactive oxygen species (ROS) responsive polymer-drug conjugate nanoparticles (DGA) to manipulate microglia polarization by suppressing the translocation of nuclear HMGB1. DGA presented excellent therapeutic efficacy in stroke mice, as evidenced by the reduction of infarct volume, recovery of motor function, suppressed of M1 microglia activation and enhanced M2 activation, and induction of neurogenesis. Altogether, our work demonstrates a close association between HMGB1 and microglia polarization, suggesting potential strategies for coping with inflammatory microglia-related diseases.

## Highlights

• We synthesized GA-boronate ester-conjugated diethylaminoethylen-dextran polymer-drug conjugate nanoparticles.
• The DGA nanoparticles achieve ROS-responsive drug release.
• The DGA nanoparticles inhibit cytoplasmic translocation of nuclear HMGB1, thus modulate microglia to M2 phenotype.
• The DGA nanoparticles effectively alleviate the pathology of stroke, reduce infarct volume, and enhance neurogenesis.

## Graphical abstract

**Figure:**
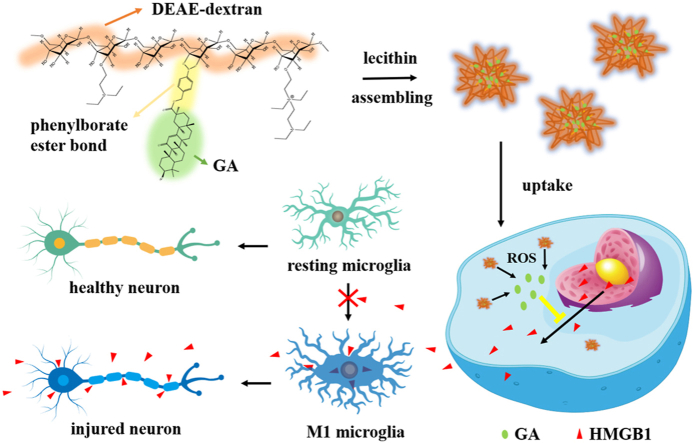

## 1 Introduction

Stroke is a cerebral vascular incident of high morbidity and mortality, of which 80% is related to cerebral ischemia [[1], [2], [3]]. Immune response is a pivotal mechanism affecting cerebral ischemia pathogenesis, pathobiology and outcomes of stroke [4,5]. In such context, the immune response is initiated by a mass of damage-associated molecular patterns (DAMPs) released from ischemic brain cells, commonly including adenosine triphosphate (ATP), high mobility group box 1 (HMGB1), reactive oxygen species (ROS), and interleukin (IL)-33 [[6], [7], [8]]. Microglia are the major resident immune cells in the brain. Carrying the pattern recognition receptors to DMAPs, they are among the first cell populations reacting to these danger signals within hours or even minutes after ischemic injury, and their actions further aggravate neuroinflammation in the peri-infarct region after ischemia [9]. However, previous evidences showed that microglia are double-edged swords in the battle for nerve repair because the resting microglia can adopt two different activation states, namely the classically activated (M1) and alternatively activated (M2) phenotypes [[10], [11], [12], [13]]. M1-like microglia activated by DAMPs play important roles in inflammation by secreting numerous proinflammatory cytokines and mediators that contribute to immune-mediated neuronal damage and impede neurogenesis. In contrast, the M2 microglia mostly conduct protective functions and confer neuroprotection by secreting anti-inflammatory cytokines and neurotrophic factors. Thus, modulating microglia activation state in ischemia would be an attractive therapeutic approach [4,11,[14], [15], [16], [17]].

HMGB1, a DNA-binding nuclear protein expressed in all nucleated animal cells, is one type of hidden DAMPs. It is involved in physiological functions including DNA replication, transcription, and repair [18]. Once transported into the cytoplasm, it can participate in immune responses [19]; when released into extracellular space, it works as a powerful mediator of inflammation and stimulates the innate immune system either alone or as part of a pro-inflammatory cascade [20]. During cerebral ischemia, HMGB1 is released from neurons, reactive microglia and reactive astrocytes upon primary injury, and then binds to specific receptors of microglia to further aggravate the secondary injury. HMGB1 altered the microglia polarization in some disease models, such as traumatic brain injury (TBI) [21] or spinal cord injury (SCI) [22]. Gao et al. found the inhibition of HMGB1 attenuated TBI at least partially by regulating microglia to M2 polarization [21]. Fan et al. demonstrated that microglia M1 activation was modulated through HMGB1-receptor for advanced glycation end-products (RAGE) pathway [22]. Thus, blocking the HMGB1-RAGE axis improved the outcome of transient middle cerebral artery occlusion mice [23]. Other receptors on microglia such as Toll-like receptors (TLRs) [24], scavenger receptor Mac1 [25], and chemokine receptor CXCR7 [20] also interact with HMGB1 and activate microglia.

Glycyrrhizic acid (GL), a triterpene glycoside derived from licorice, is a confirmed pharmacological inhibitor of HMGB1 [26]. 18β-glycyrrhetinic acid (GA), a major bioactive hydrolyzed metabolite of GL, inhibits HMGB1 effectively [27]. The inhibitory effect of GL or GA on HMGB1 is manifested in two aspects [28]: It suppresses HMGB1 phosphorylation inside the cells, thus decreasing the secretion of HMGB1. In addition, it inhibits the overall expression of HMGB1 as well. However, GA has poor bioavailability due to lipophilicity, low solubility, and short biological half-time, which hinders its clinical application [29]. In addition, GA is found to induce apoptosis by increasing the production of ROS [30], which is dose-dependent, indicating potential toxicity of GA when overdosed. Moreover, chronic consumption of large doses of GA will cause hyponatremia, hyperkalemia, hypertension and hyperglycemia [31]. Therefore, a controlled release system is critical for its therapeutic application.

The advances of drug delivery systems (DDSs) have provided new routes for improving the efficiency of drug delivery [[32], [33], [34], [35]]. To reach effective concentration while reducing the sides effects of GA, Lu et al. designed methoxy poly(ethyleneglycol)-poly(d,l-lactic acid) (mPEG-PLA) block copolymers to enhance its solubility. Compared with GA in conventional adjuvant, GA in mPEG-PLA hybrid nanoparticles had 2.75 times in area under curve (AUC), 1.7 times in mean residence time (MRT) within 8 h, and thus prolonged circulation time in blood after single dose intravenous injection [36]. Bovine serum albumin (BSA), an excellent natural polymer with biocompatibility, were also used to improve the utilization of GA [29]. However, the therapeutic outcome of traditional DDSs is usually limited by non-specific drug release and rapid plasma elimination. Therefore, it is crucial to design stimuli-responsive DDSs which can release drugs in specific site where endogenous disease-associated stimuli exist [37,38] or exogenous signals are applied [39,40]. For instance, Jaglal et al. co-delivered vancomycin and GA via pH-responsive lipid-polyallylamine hybrid nanoparticles for enhancing drug delivery and antibacterial activity [41].

ROS are endogenous stimulus signals and are overproduced during ischemia [42]. Emerging ROS-sensitive materials have presented great potential for intelligent DDSs [43,44]. Lv et al. developed a ROS-responsive boronic ester conjugated dextran for delivery of a neuroprotective agent NR2B9C against ischemic stroke [45]. Lu et al. prepared an amphiphilic polymer with fibrin-binding pentapeptide functionalized hydrophilic poly(ethylene glycol) (PEG) and hydrophobic phenylboronic ester-modified polylysine [14]. Rapamycin was encapsulated in ROS-responsive polymers to achieve controlled drug release in the ischemic site. Zhang et al. designed pH/ROS dual-responsive nanoparticles for drug delivery [46]. The acetalated β-cyclodextrin (β-CD) was sensitive to pH change and the 4-phenylboronic acid pinacol ester modified β-CD was easily hydrolyzed under 1 mM H_2_O_2_.

**Scheme 1:**
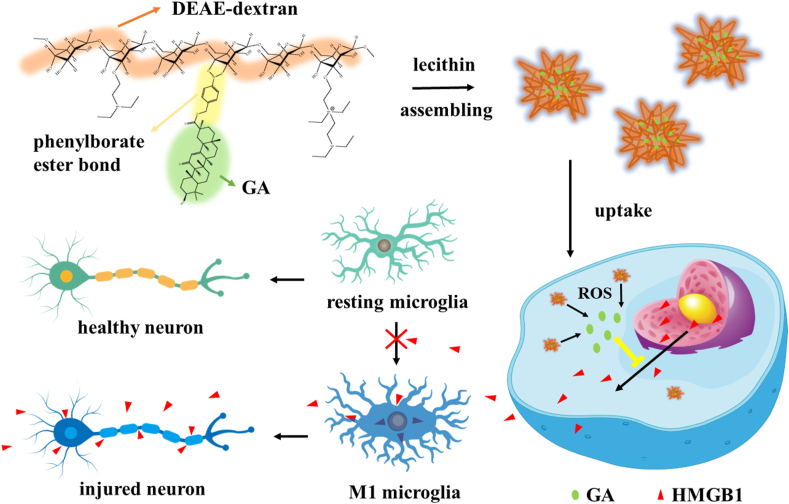
ROS-responsive drug-conjugated nanoparticles inhibit HMGB1 and regulate microglia polarization.

Here, we synthesized ROS-responsive GA-conjugated diethylaminoethylen (DEAE)-dextran nanoparticles (DGA) to manipulate microglia polarization for improving the efficacy of cerebral ischemia therapy (Scheme 1). Based on the elevated levels of ROS in the ischemic site, boronate ester, a widely used ROS-responsive drug conjugating linker [47], was incorporated in polymeric nanoparticles, which efficiently controlled drug release under ROS stimulation. GA, as an effective hydrophobic intracellular inhibitor of HMGB1, could inhibit the intracellular translocation of nuclear HMGB1, thus reducing the pro-inflammatory effects of HMGB1 on downstream cells. The hydrophilic backbone DEAE-dextran increased the water solubility of GA [48]. Furthermore, the lecithin was introduced to improve the stability of nanoparticles. Our results suggested that the polymer-drug conjugate nanoparticles could effectively alleviate the pathology of stroke, reduce infarct volume, and enhance neurogenesis, through inhibiting HMGB1 translocation and switching microglia M1 or M2 phenotype.

## 2 Materials and methods

### 2.1 Chemicals

2-diethylaminoethylen-dextran (DEAE-Dex, Mw = 70 kDa) was purchased from TdB Labs, Sweden. 18β-glycyrrhetinic acid (GA) and Nile red (NR) were purchased from Aladdin. 4-(bromomethyl)benzeneboronic acid pinacol ester (BBA) and 1,8-diazabicyclo[5.4.0]undec-7-ene (DBU) were obtained from Macklin, China. Anhydrous dimethyl sulfoxide (DMSO) and anhydrous N,N-dimethylformamide (DMF) were obtained from J&K Scientific, China. Deuterated chloroform (CDCl_3_) and DMSO-*d6* were purchased from Innochem, China. Polystyrene-boronic acid and 4′,6-diamidino-2-phenylindole (DAPI) were purchased from Sigma-Aldrich, USA. Lecithin and 2% 2,3,5-triphenyltetrazolium chloride (TTC) were purchased from Shanghai yuanye Bio-Technology Co., Ltd., China. Rose Bengal and lipopolysaccharide (LPS) were gained from Sigma, USA. BV2 cells (CL-0493) were purchased from Procell, China. Dulbecco's modified Eagle's medium (DMEM), fetal bovine serum (FBS) and penicillin/streptomycin were gained from Gibco, USA. Cell counting kit-8 (CCK-8) was purchased from Target Molecule Corp, USA. Anti-HMGB1 antibody (ab18256), anti-Iba1 antibody (ab283319), anti-CD16/32 antibody (ab223200), anti-CD206 antibody (ab64693), anti-NeuN antibody (ab177487), anti-Tuj1 antibody (ab18207), goat polyclonal secondary antibodies to rabbit IgG-H&L (Alexa Fluor® 488, ab150077 and Alexa Fluor® 647, ab150079) were purchased from Abcam, UK. The enzyme-linked immunosorbent assay (ELISA) kit of HMGB1 was supplied by Beijing Solarbio Science & Technology Co., Ltd., China. Unless otherwise noted, organic solvents and inorganic salts were purchased from Sinopharm Chemical Reagent Co., Ltd., China, and used without further purification. Water in all the experiments was purified using a Millipore-Q water-purification System (Milli-Q Integral 3, Millipore, USA).

### 2.2 Synthesis and characterization of compound 1

The chemical reaction of BBA-conjugated GA was prepared as previously reported with some modifications [49,50]. 485.0 mg GA, 455.0 mg BBA, and 276.0 mg K_2_CO_3_ were dissolved in 10 mL anhydrous DMF. The reaction solution was agitated at 80 °C for 12 h and then cooled with 100 mL ice water after reaction. The crude products were easily recycled from the system as sediments, which were washed several times using water, dissolved with dichloromethane (DCM), and dried with MgSO_4_. The products were purified by column chromatography with DCM and ethyl acetate (EA) (5:1, v/v). A white solid was obtained as compound 1 which was further confirmed by ^1^H nuclear magnetic resonance (NMR, Avance-400, Bruker, Switzerland) and electrospray ionization (ESI, GCT Premier GC-TOFMA, Waters, USA).

### 2.3 Synthesis and characterization of compound 2

Pinacolyl boronate esters can be deprotected by transesterification with polystyrene-boronic acid [51]. 485.0 mg compound 1 and 3.5 g polystyrene-boronic acid were added into 30 mL of tetrahydrofuran (THF): 1 M hydrochloric acid (HCl) = 9 : 1 solution under stirring for 72 h at room temperature. The suspension was filtered and the filtrate was collected. The pH of filtrate was adjusted to 7.0 with saturated NaHCO_3_ solution. THF was removed under reduced pressure, and the solution was extracted with DCM and washed by water. The organic layer was collected and dried using MgSO_4_. The crude products were purified by column chromatography with gradient elution consisting DCM and EA from 5 : 1 to 1 : 2. After removing the solvent, a light yellow solid was obtained as compound 2 which was further confirmed by ^1^H NMR, ^13^C NMR (100 MHz, Bruker, Switzerland) and ESI.

### 2.4 Synthesis and characterization of GA-conjugated DEAE-dextran (GA-Dex)

Phenyl boronic acid can react with 1,2-cis-diols of dextran to form boronic esters [47,52]. 153.0 mg compound 2 and 70.0 mg DEAE-Dex were dissolved in 10 mL anhydrous DMSO. 20 μL DBU was added to the solution and the mixture was stirred overnight at room temperature under the protection of N_2_. After reaction, the polymer solution was precipitated into EA and the collected sediments were washed three times with EA and once with water. A white product was obtained by drying overnight under vacuum. The structure was identified by ^1^H NMR and Fourier transform infrared (FTIR, Nicolet 6700, Thermo Fisher Scientific, USA). The grafting efficiency of GA was measured by ultraviolet spectrometer (UV, UV2600, Shimadzu, Japan).

### 2.5 Preparation and characterization of lecithin coated GA-Dex nanoparticles (DGA)

The DGA were formed by self-assembly strategy [45]. Briefly, 1 mg/mL GA-Dex and 2.5 mg/mL lecithin were fully ultrasonic using a sonicator (KQ-400KDE, Kunshan ultrasonic instrument Co., Ltd, China), respectively. The DGA nanoparticles were obtained by ultrasound at 400 W for 10 min after mixing 1 mg/mL GA-Dex and 2.5 mg/mL lecithin at volume 1 : 1. The hydrodynamic diameter and zeta potential were determined by dynamic light scattering (DLS, Zetasizer 3000, Malvern, USA). The morphologies were observed under transmission electron microscope (TEM, HT-7700, Hitachi, Japan).

### 2.6 Stability and H_2_O_2_-responsive GA release of DGA

1.8% NaCl solution was mixed with equal volume of DGA solution. The hydrodynamic diameters of nanoparticles at day 0, 1, and 7 were detected by DLS. H_2_O_2_-triggered hydrolysis was performed in saline containing 1 mM H_2_O_2_ at 37 °C. DLS and turbidity change images were used to illustrate the hydrolysis process. High performance liquid chromatography (HPLC, 1260Ⅱ, Agilent Technologies, USA) method was applied to determine the content of released GA triggered by 1 mM H_2_O_2_. 350 μg/mL DGA solution containing 0 or 1 mM H_2_O_2_ was set in 37 °C shaker. After 0, 0.5, 1, 2, 4 6, 8, 10 h co-incubation, the solution was centrifuged with Millipore tube (MACO = 3.5 kDa) for 0.5 h and the supernatant was tested by HPLC. The standard curve of GA was obtained at the same time. A reverse-phase column (C18, 150 × 4.6 mm, 3.5 μm) and an ultraviolet detector (250 nm) were used. The mobile phase was made of methanol: water (pH of 3, adjusted by HCl) = 90 : 10 (v/v), with 1 mL/min flow rate and 20 μL injection volume.

### 2.7 Cytotoxicity and cell uptake *in vitro*

BV2 cells were cultured in DMEM with 10% (v/v) FBS, 100 U/mL penicillin, and 100 μg/mL streptomycin at 37 °C in a 5% CO_2_ incubator. The cytotoxicity of DGA against BV2 cells was analyzed by CCK-8 assay. Briefly, BV2 cells were seeded in a 96-well plate at a density of 1.0 × 10^4^ cells/well and incubated over 6 h to allow cell adherence. After incubation with a series of concentration of DGA for 24 h, the cells were washed once with PBS and incubated for another 1 h with CCK-8. Absorbance at 450 nm was measured using microplate reader (Infinite M200, TECAN, Switzerland). Cells treated with saline were used as control (denoted as 100% viability).

Nile red dye loaded liposomes (NR@Lip, synthesis method was in supporting information) were used as nanoparticles for cell uptake experiments. Firstly, BV2 cells were seeded in a 6-well plate at a density of 1.0 × 10^6^ cells/well overnight in 37 °C incubator. Then, 1.0 μg/mL LPS was used to induce M1-like activation for 12 h. The untreated cells remained resting state were set as control. 50 μg/mL NR@Lip were administered and incubated with cells for 1, 2, and 4 h at 37 °C. After incubation, the cells were washed twice with PBS and collected. The cellular fluorescence was determined by flow cytometer (FACS caliber, BD Biosciences, USA). Data were analyzed using the FlowJo software. For confocal images, BV2 cells were seeded in 35 mm confocal dishes at a density of 1.0 × 10^6^ cells/dish overnight in 37 °C incubator. The following treatment before cell collection was the same as described above. Instead of collecting cells, the cells were treated with 4% paraformaldehyde solution and stained by DAPI. All images were obtained using confocal microscopy (Leica TCS SPE, Leica, Germany) and analyzed using LAS-AF-Lite software.

### 2.8 Inhibition of HMGB1 translocation and suppression polarization toward the M1 phenotype *in vitro*

BV2 cells were seeded in 35 mm confocal dishes at a density of 1.0 × 10^6^ cells/dish and proliferated until adherent growth occurred. 1.0 μg/mL LPS was used to simulate inflammatory conditions for 12 h, and then treated LPS-induced BV2 cells with saline, 0.2 μg/mL GA, 0.8 μg/mL Dex +2.5 μg/mL lecithin, 3.5 μg/mL DGA for 12 h, respectively. Then, the samples were washed with PBS, treated with 4% paraformaldehyde, blocked with 5% BSA, permeated with 0.2% Triton X-100, treated with primary antibody (anti-HMGB1 or anti-CD206 or anti-CD16/32) and secondary antibody (Alexa Fluor® 488 or Alexa Fluor® 647) according to the manufacturer's protocol. Finally, DAPI was used to label cell nucleus. All images were obtained using confocal microscopy and analyzed using LAS-AF-Lite software. Cells without LPS simulation were set as control. The concentration of nuclear HMGB1 level was quantified using ELISA kit according to the manufacturer's instructions. Besides, flow cytometry was conducted for quantification of phenotypic associated proteins. Briefly, BV2 cells were seeded in 6-well plates at a density of 1.0 × 10^6^ cells/well. After cell adherence, the cells were treated the same way as before. Finally, the cells were harvested for flow cytometry. Data were analyzed using the FlowJo software.

### 2.9 Photochemically induced cerebral infarction model

All surgical procedures and postoperative care were performed in accordance with guidelines of Animal Care and Use, Zhejiang Academy of Medical Sciences (Approval No. ZJCLA-IACUC-20020012). Male C57BL/6 mice (7–8 weeks old) weighing 18–22 g in this experiment were maintained on a 12 h light/dark cycle.

Focal cerebral thrombosis was induced by photothrombosis [53]. Mice were anesthetized with 2% isoflurane and maintained with 1% isoflurane in an oxygen/air mixture by using a gas anesthesia mask in a stereotaxic frame. 1% (w/w) Rose Bengal was infused intravenously at a dosage of 30 mg/kg via 5 minutes before illumination. Scalp hairs of mice were shaved, and the scalps were cut open and periosteum was removed. The infarction lesion was formed by irradiating the right sensorimotor cortex [54] which was located at medial-lateral (M/L): +2.0 mm and anterior-posterior (A/P): 0.0 mm. For illumination, a 532 nm green laser (LR-GSP-532/100 mW, Changchun Laser Technology Co., Ltd, China) with a 1.0-mm aperture was applied with power of 25 mW for 10 min. The scalps were sutured at the end of experiments. Mice were placed at 37 °C homeothermic blanket until woke up.

### 2.10 Behavioral tests

Stroke mice were randomly assigned to four groups: (1) saline, (2) 40 μg/mL Dex + 125 μg/mL lecithin, (3) 10 μg/mL GA, (4) 175 μg/mL DGA. After thrombosis, a micro-syringe with 31 G needle was located at M/L: + 1.0 mm, A/P: + 0.5 mm for intracerebroventricular injection [55]. The injection depth was 2.5 mm, and the injection rate was 0.2 μL/min for 10 min (2 μL in total). The needle was removed after remaining for 5 min.

Foot fault test [56] was performed at 1, 3, and 7 days after therapy. Mice were placed in a suspended steel grid with 1.2 cm × 1.2 cm rectangular holes. Without external interference, the movements of mice were video-recorded for 5 min. When the affected paw (contralateral to injury hemisphere) entirely slipped through or missed the grid, it was considered an individual foot fault. Number of foot fault errors in the first total 100 steps in video was counted.

Cylinder test is another sensitive analysis to predict forelimb motor function of stroke [53,57]. Mice were placed in a transparent 500 mL beaker. Mirrors were placed behind the cylinder to record forelimb movements from all directions. Criteria for quantifying cylinder behavior scores referred to previous study [16]. A video was recorded at 7 days post-treatment until a mouse performed at least 20 movements. The final score = (nonimpaired forelimb movement - impaired forelimb movement)/(nonimpaired forelimb movement + impaired forelimb movement + both movement). A lower score suggests better motor function.

### 2.11 Cerebral infarct volume evaluation

After 7 days treatment, all mice were neck breaking and the brains were collected. For TTC staining (n = 3 for each group), the brains were cooled at −20 °C for 5 min and sectioned coronally into 6 slices. Whereafter, the slices were stained with 2% TTC at 37 °C for 20 min [58]. The cerebral infarct areas were unstained, which showed white, while the normal tissues were stained red. The results were presented in the form of digital photos. Cerebral infarct volume was furthered calculated using Image J.

### 2.12 Immunohistochemistry of HMGB1 in brain sections

The brains (n = 3 for each group) were fixed in 4% paraformaldehyde for 24 h, dehydrated in graded ethanol, embedded in paraffin, and cut into 5 μm-thick sections for histological assay. Brain sections were deparaffinized in xylene, rehydrated through decreasing concentrations of ethanol, and washed in PBS. HMGB1 immunohistochemical staining [21] were conducted according to a standard protocol. The total number of cells with or without nuclear HMGB1 were calculated using Image J. The nuclear localization proportion of HMGB1 was calculated as the number of cells with HMGB1 in nucleus/the total number of cells × 100%.

### 2.13 Immunofluorescence of microglia phenotype-related markers in brain sections

Before staining, the paraffin sections were deparaffinized, rehydrated, washed, permeabilized and blocked. Primary antibodies: anti-Iba1 antibody for microglia, anti-CD16/32 antibody for M1 microglia, anti-CD206 antibody for M2 microglia, anti-NeuN antibody and anti-Tuj1 for neurons [59]. The brain sections were incubated with primary antibodies at 4 °C overnight, washed with PBS and labelled with fluorescently labelled secondary antibody for 2 h at room temperature. The acquired sections were washed, and then stained nucleus with DAPI for 5 min. Finally, slides were imaged by slide scanner system (Axio Scan. Z1, Zeiss, Germany) and analyzed by CaseViewer.

### 2.14 Biosafety *in vivo*

Healthy mice were divided into three groups: (1) healthy, (2) intracerebroventricular injection (ICV) of 2 μL 175 μg/mL DGA, and (3) intravenous injection (IV) of 100 μL 17.5 μg/mL DGA. *In vivo* toxicity was analyzed including body weight, blood routine, blood biochemistry, and histopathological tests. At day 1 and day 7 post-injection, blood and main organs, including heart, liver, spleen, lung, kidney, brain were collected. Alanine aminotransferase (ALT), aspartate aminotransferase (AST), blood urea nitrogen (BUN), and creatinine (CRE) were conducted upon blood collection as well as red blood cell (RBC), white blood cells (WBCs), platelets (PLT) and neutrophil (Neu). Main organs were analyzed using hematoxylin-eosin (H&E) staining.

### 2.15 Statistical analysis

Experimental data were provided as mean ± standard error of the mean (SEM). The significant differences among groups were analyzed using one-way analysis of variance (ANOVA) in Origin software. *P* < 0.05 was considered statistically significant.

## 3 Results and discussion

### 3.1 Synthesis and characterization of the GA-Dex polymer

**Fig. 1:**
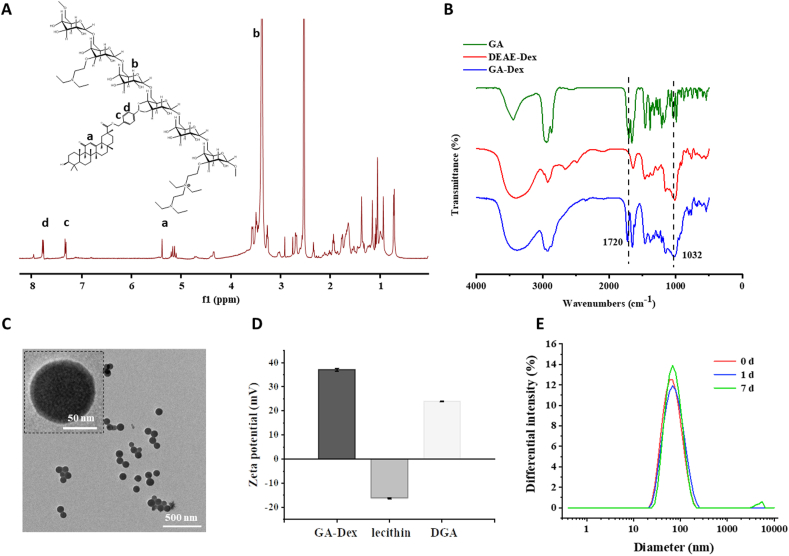
**The chemical structure of GA-Dex polymer and characterization of DGA nanoparticles.** (A) ^1^H NMR spectra and (B) FTIR spectra of synthesized GA-Dex. (C) TEM images of DGA freeze-dried in water; scale bar = 500 nm (embedded scale bar = 50 nm). (D) Zeta potentials of GA-Dex, lecithin, and DGA in water (n = 3). (E) Average hydrodynamic diameter of DGA in 0.9% NaCl at day 0, 1, and 7, respectively.

Firstly, BBA-conjugated GA was prepared. The carboxylic acid groups on GA could be converted to potassium salts under alkaline conditions of K_2_CO_3_, which acted as nucleophiles and reacted with bromine groups on BBA to form esters (compound 1, Fig. S1). After that, the boronic acid pinacol ester groups of compound 1 were deprotected by transesterification with polystyrene-boronic acid to obtained compound 2 (compound 2, Fig. S2). In order to conjugate compound 2 onto the DEAE-dextran, a coupling reaction was applied between boronic acid on compound 2 and the diol on dextran. In the ^1^H NMR spectrum of GA-Dex (Fig. 1A), the multiple signal peaks from 3.0 ppm to 4.0 ppm belonged to glucose unit, in accordance to the ^1^H NMR of DEAE-dextran (Fig. S3). Doublet at 7.3 ppm and 7.7 ppm were suggested the phenyl groups in conjugated BBA part and the multiple peaks from 0.5 ppm to 2.0 ppm were corresponded to the alkyl structure in conjugated GA part, which proved the successful synthesis of GA-Dex. Furthermore, FTIR confirmed the successful grafting of GA as well (Fig. 1B). The sharp peak at 1720 cm^−1^ represented the characteristic stretching vibrational absorption of carbanyl groups on GA while the strong and wide peak at 1032 cm^−1^ represented the stretching vibration absorption of abundant carbon-oxygen single bonds on dextran. The GA conjugation ratio was 21.86 wt% which was calculated using UV spectroradiometer (Fig. S4).

### 3.2 Preparation and characterization of lecithin coated GA-Dex nanoparticles (DGA)

Amphiphilic GA-Dex self-assembled into spherical particles (Fig. S5A) with a diameter of about 531.2 nm (PDI = 0.149) in water (Fig. S5B). However, the particles were not stable in saline. The particle size become larger (615.1 nm, PDI = 0.266) (Fig. S5B) as soon as they dispersed into saline and obvious precipitations were observed later (not shown). Therefore, lecithin was added to improve the stability of GA-Dex nanoparticles. By adjusting the ratio of GA-Dex and lecithin, the particle size decreased to 104.8 nm (Figs. S5D and E), as a result of insertion of the amphiphilic phospholipid layer, which made the particles more tightly entangled by hydrophobic interaction. But when the GA-Dex/lecithin ratio increased to 1 : 3, a peak of micron size appeared (Fig. S5F), which likely resulted from excess lecithin referring to the lecithin peak (Fig. S5C). Consequently, 1 : 2.5 ratio of GA-Dex and lecithin was chosen for following study. As shown in Fig. 1C, the DGA were homogeneous spherical nanoparticles. The zeta potential of DGA was between electropositive GA-Dex and electronegative lecithin, which indicated the effective integration of those components (Fig. 1D). Afterward, we investigated the stability of DGA nanoparticles in saline for 7 days. Little change in DGA size (Fig. 1E) was observed, indicating excellent stability.

### 3.3 H_2_O_2_-responsive GA release of DGA

**Fig. 2:**
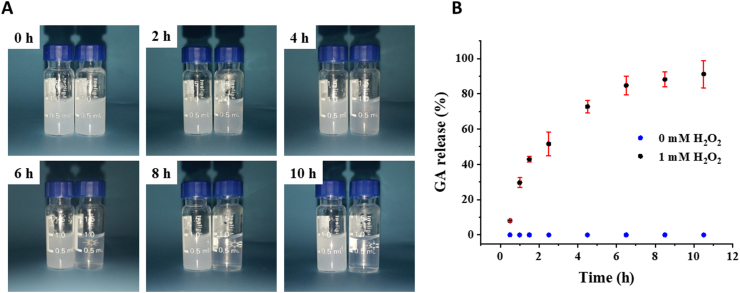
**H**_**2**_**O**_**2**_**-responsiveness of DGA.** (A) Digital photos of DGA in saline with (right bottle)/without (left bottle) 1 mM H_2_O_2_ at 37 °C for a series of co-incubation time. (B) Accumulated GA release from DGA in saline in the presence of 1 mM H_2_O_2_. The GA signal was monitored using HPLC (n = 3). The release amount was calculated based on a standard curve of GA.

As ROS are overexpressed in cells and tissues of pathological conditions, boronic acids have been incorporated into drug delivery systems as a ROS stimuli-responsive functional part [60,61]. We suggested that phenyl borate motifs, as the linkage between GA and dextran, could be broken to release chemically conjugated GA in oxidative environment such as high concentration of H_2_O_2_. The H_2_O_2_ reactivity of DGA was explored in saline containing 1 mM H_2_O_2_ monitored by DLS (Fig. S6A). The size increased with incubation time until a micron-sized aggregate formed. Moreover, the higher H_2_O_2_ concentration (10 mM), the faster particle size increase (Fig. S6B). In addition, digital images were recorded to visualize the process of hydrolysis of DGA with 1 mM H_2_O_2_ at 37 °C. The turbidity of solution without H_2_O_2_ could still be observed opaque after 10 h incubation while the solution with H_2_O_2_ gradually became clear (Fig. 2A). Furthermore, simultaneous GA release was analyzed by HPLC. As shown in Fig. 2B, nearly 50% of the GA was released within 2.5 h under 1 mM H_2_O_2_ stimulation. The plateau was almost reached at 6.5 h and totally 91.18% of GA was released within 10.5 h. DGA without H_2_O_2_ trigger showed no GA release. Compared with other ROS responsive phenylboronic acid-conjugated materials whose response range of H_2_O_2_ were roughly 0.1 mM–5 mM [45,[62], [63], [64], [65]], the H_2_O_2_ sensitivity of DGA prepared by us was consistent with that reported in literature. In summary, DGA could respond to 1 mM H_2_O_2_ to release drugs.

### 3.4 Cytotoxicity and cell uptake *in vitro*

**Fig. 3:**
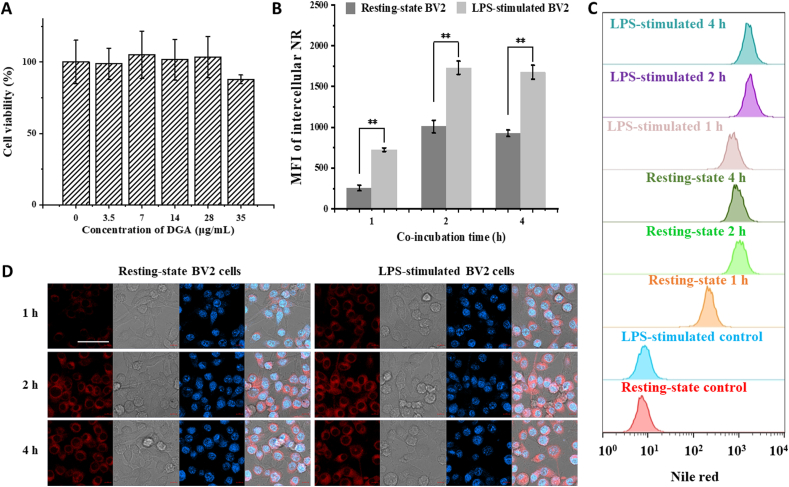
**Biocompatibility of DGA and the phagocytosis of BV2 cells.** (A) Cell viability of BV2 cells incubated with different concentrations of DGA for 24 h (n = 3). (B) Quantified analysis of NR@Lip in BV2 cells with flow cytometry by increasing of co-incubation time (n = 3). The mean fluorescence intensity (MFI) was relative to the cells without NR@Lip addition. (C) Typical flow cytometry data of cellular uptake of NR@Lip in BV2 cells. (D) Confocal images of intracellular NR@Lip (red) in BV2 cells (blue). Nuclei stained by DAPI. The gray images were bright field images from which cell outlines could be observed; scale bar = 50 μm. Data are presented as mean ± SD. **p < 0.01.

BV2 is a murine-microglia-derived cell line that largely possesses the morphological, phenotypic and functional characteristics of primary microglia, and has been widely used in neurological studies [11]. Therefore, BV2 cells were chosen as the model for subsequent experiments. First, the biosafety of DGA *in vitro* was examined. When DGA concentration was less than 35 μg/mL, the viability of BV2 cells measured by CCK8 assay was more than 85% of the control after 24 h co-incubation (Fig. 3A). Since microglia are inherent phagocytes in the CNS, and their phagocytic activity can be altered by different activation [66]. To authenticate the different phagocytosis of resting-state or M1-state BV2 cells, cell uptake was tracked with fluorescent molecules labelled particles (NR@Lip). The leakage of loaded NR dye was less than 0.6% in PBS, aCSF, and PBS containing 10% FBS, measured by fluorescence spectrometer (Fig. S7), indicating almost no leakage of fluorescent dye during the experiment. As shown in Fig. 3B–D, both of the confocal images and the flow cytometry results indicated that the LPS-stimulated M1-state BV2 cells had a stronger phagocytic ability than resting cells at 1, 2, and 4 h after co-incubation. However, there was no significant change in cell uptake at 2 h and 4 h, indicating that cellular uptake had reached a plateau in a short period. We also tested the cell uptake of IL4-stimulated BV2 cells which were considered as M2 phenotype. As shown in Fig. S8, BV2 cells with M1 phenotype had a stronger phagocytosis of particles than M2 phenotype BV2 cells, and ingested nearly twice as much. The particle intake of M2-state BV2 cells was greater than that of resting-state cells within 1 h, but was equal to that of resting-state cells after 2 h. Our result was consistent with the studies of Siddiqui et al. [67] that M1 phenotype cells might have a higher phagocytic capacity. These results suggested that M1-polarized microglia might increase uptake of DGA nanoparticles, thus further enhancing the effect of DGA on those cells.

### 3.5 Effect of DGA on inhibition of HMGB1 translocation and transformation of microglia polarization *in vitro*

**Fig. 4:**
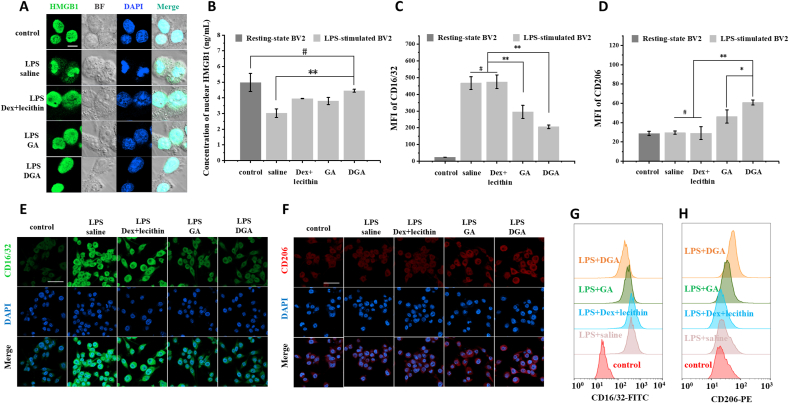
**Inhibition of HMGB1 translocation and transformation of microglia phenotype *in vitro*.** (A) Cellular immunofluorescence images of HMGB1 after 1 μg/mL LPS stimulation for 12 h, followed by saline, 0.2 μg/mL GA, 0.8 μg/mL Dex + 2.5 μg/mL lecithin, 3.5 μg/mL DGA for 12 h, respectively. Untreated resting-state BV2 cells were set as control. HMGB1 was stained green and nuclei were stained blue. The gray images were bright field images from which cell outlines could be observed. Scar bar = 10 μm. (B) The concentration of nuclear HMGB1 after treatment (n = 3). (C–H) Represented immunofluorescence staining images of M1 phenotype marker CD16/32 (E, green) and M2 phenotype marker CD206 (F, red) in BV2 cells. Nuclei were counterstained with DAPI (blue); scar bar = 50 μm. Quantification of CD16/32 (C) and CD206 (D) by flow cytometry (n = 3). Typical flow cytometry data of CD16/32 (G) and CD206 (H) among different treatments. Data are presented as the mean ± SD. *P < 0.05, **P < 0.01, # represented no significant difference.

Neuroinflammation *in vitro* were induced by LPS, characterized by enhanced production of ROS, HMGB1, and other inflammation-related factors [68,69], creating an optimal inflammatory milieu for validating the neuroprotective effects of therapeutic drugs. HMGB1 is a chromatin-associated protein located in the nucleus, which functions as an important proinflammatory factor once it is released into the extracellular environment. Before that, HMGB1 needs to be translocated from nucleus to cytosol. Therefore, immunofluorescence was used to observe the translocation of HMGB1. BV2 cells were stimulated by LPS and treated with saline, a mixture of DEAE-Dex and lecithin (group Dex + lecithin), GA, and DGA, respectively. Untreated resting-state BV2 cells were set as control. As shown in Fig. 4A and Fig. S9A, under saline treatment, the HMGB1 was transferred from nucleus to cytoplasm, resulting in less HMGB1 in the nucleus. In GA or DGA group, HMGB1 was largely confined to the nucleus. This phenomenon was consistent with the pharmacological effects of GA on HMGB1 release [28,70]. As long as HMGB1 was not released, its proinflammatory effect on downstream cells could be attenuated. We further quantified the nuclear HMGB1 by ELISA kit. As shown in Fig. 4B, HMGB1 was lost a lot in the nucleus after LPS stimulation and saline treatment while HMGB1 was retained in the nucleus in the DGA group.

Microglia are extremely sensitive and may alter their quiescent state according to the extracellular environment. To investigate the effect of DGA on BV2 cells polarization, LPS was used to induce M1 phenotype and mimic the inflammatory environment as previously described. As shown in Fig. 4C,E,G, confocal images and flow cytometry results both showed that the LPS-stimulated BV2 cells with saline or Dex + lecithin treatment had a higher expression of M1 phenotype marker CD16/32 than those with GA or DGA treatment. The mean fluorescence intensity (MFI) of CD16/32 in GA or DGA group decreased by 36.8% or 56.0% compared with saline group, respectively. The expression of CD206 in the DGA group was two folds of the saline group and 1.3 times of the GA group (Fig. 4D,F,H). The positive control (IL-4 treated group) was displayed in Figs. S9B and C. Collectively, these data indicated that DGA nanoparticles attenuated the M1-type polarization of BV2 cells and induced BV2 cells into M2-like phenotype.

### 3.6 Effect of DGA on infarct volume and locomotion recovery

**Fig. 5:**
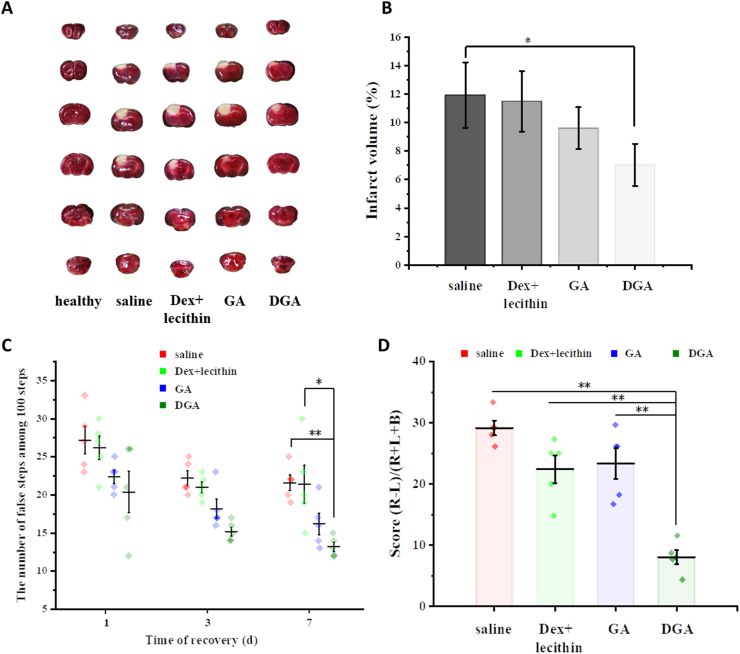
**Effect of DGA on brain infarct volume and motor functional recovery after stroke.** (A) Representative images of the TTC-stained brain slices after saline, 40 μg/mL Dex + 125 μg/mL lecithin, 10 μg/mL GA, 175 μg/mL DGA treatments after 7 d post-stroke. The normal tissue was stained red while injured tissue was unstained white. (B) Infarct volume of the brains after different treatments at 7 d post-stroke (n = 3). (C) Ethology studies of foot fault test at day 1, 3, and 7 after therapy (n = 5). (D) cylinder test at day 7 after therapy (n = 5). *P < 0.05, **P < 0.01.

Photothrombotic infarction is a reliable and highly reproducible animal model due to the thrombo-embolism of photosensitive dyes at a fixed light location [53]. The size of the infarct can be easily controlled by the duration or intensity of the irradiation. In addition, photothrombotic model is also widely used for the study of potential neuroprotective agents of stroke [71]. Therefore, photochemically induced cerebral infarction model was used to substantiate our strategy. Stroke mice were randomly divided into four groups and intracerebroventricularly injected immediately with saline, 40 μg/mL Dex +125 μg/mL lecithin, 10 μg/mL GA, 175 μg/mL DGA (containing 10 μg/mL released GA). Mice typically lost body weight in the first few days post-injury (Fig. S10A). Behavioral tests in response to ischemic injury were examined 1, 3, and 7 days after different treatments and the infarct volumes were stained with 2% TTC 7 days after treatment. As shown in Fig. 5A and B, the infarction areas (white) of DGA group reached 7.0% of the total brain sections compared with 11.9% in the saline group or 9.6% in the GA group, suggested the DGA had better protective efficiency compared with equivalent dose of GA solution. Stroke also leads to limb-use deficits in mice which are prone to stomp out on the grid. Consistent with the results of TTC staining, the number of foot fault taken on the grid was significantly reduced in DGA group although spontaneous recovery occurred in mice to some extent. Compared to the saline group at day 1 and day 7, DGA group reached a 25% reduction at day 1 post-therapy and 38.9% reduction at day 7 post-therapy (Fig. 5C). The cylinder test also provides a quantitative and objective assessment method of dexterity of contralateral forelimb in stroke mice. The stroke mice showed significant asymmetry in forelimb use, with a tendency to use the unaffected forelimb to touch or support the cylinder wall. As shown in Fig. 5D, there was a significant improvement in DGA-treated group compared with all other groups after 7 days. No significant difference was found between the saline group and Dex + lecithin group in TTC staining and behavioral tests, suggested that DEAE-Dex and lecithin did not play protective roles in this treatment. It was GA that acted an active ingredient, which could reduce the brain tissue damage and protect the salvageable ischemic penumbra. Furthermore, after GA was integrated into a ROS-responsive controlled release system, its therapeutic effect was further enhanced. Collectively, these results proved strong evidences that DGA could attenuate tissue necrosis and promote motor functional recovery in stroke.

### 3.7 Effect of DGA on inhibition of HMGB1 translocation, transformation of microglia polarization and nerve regeneration *in vivo*

**Fig. 6:**
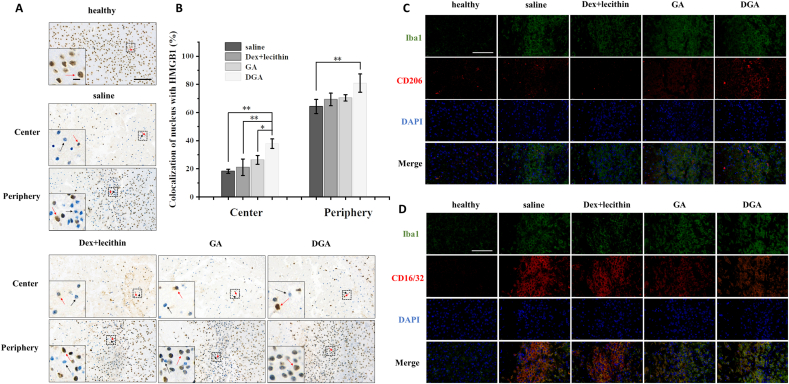
**Inhibition of HMGB1 release and transformation of microglia phenotype *in vivo*.** (A) Nuclear localization of HMGB1 detected using immunohistochemical staining at center and periphery of injury site in brain tissues. The red arrows indicated HMGB1-positive cells (healthy) and the black arrows indicated HMGB1-negative cells (injury); scale bar = 100 μm (embedded scale bar = 10 μm). (B) Quantitative nuclear localization percentage of HMGB1 at center and periphery of injury site in brain tissues (n = 3). (C) Immunofluorescence of CD206 (M2 marker, red) and (D) CD16/32 (M1 marker, red) of BV2 cells at periphery of injury site. Iba1 (microglia marker) were stained green and nuclei were stained blue; scale bar = 100 μm. On the left, center, and right of each image were lesion, boundary, and normal tissue. *P < 0.05, **P < 0.01.

HMGB1 is a DNA-binding protein located in the nucleus. Immunohistochemistry results of HMGB1 in brain sections were similar to those of immunofluorescence *in vitro*. HMGB1 co-located with nucleus in healthy cells (Fig. 6A, red arrow, Fig. S10C). When cells suffered ischemia hypoxia injury, HMGB1 was almost absent from nucleus in the center of injured areas in the saline and Dex + lecithin group (Fig. 6A, black arrow). The degree of missing intranuclear HMGB1 can reflect the degree of injury. In contrast, HMGB1 of GA or DGA group was still found in a number of nuclei in the center of injury and the nuclear localization proportion of HMGB1 in DGA group increased by about two-folds that of the saline group (Fig. 6B). However, the center of ischemia injury is usually considered irreversible while ischemic penumbra is considered salvageable [72]. Therefore, typical HMGB1 immunohistochemistry images at periphery of the infarct were displayed as well. On the left, center, and right of each image were lesions, boundary, and normal tissues, respectively. There were still many HMGB1-free cells (black arrow) in the injury boundary of saline group. The percentage of cells with nuclear HMGB1 in damaged boundary, which occupied one third of the image areas, was calculated in different groups (Fig. 6B). Portion of cells with nuclear HMGB1 at peripheral injury were 64.3% in saline group, 69.3% in Dex + lecithin group, 70.5% in GA group, and 80.9% in DGA group, suggesting that treatment with DGA could effectively inhibit the release of HMGB1, thus reducing subsequent neuroinflammation.

Microglia activation is a hallmark of neuroinflammation in the processes of cerebral ischemia. The polarization toward M1 or M2 phenotype had distinct effects. Studies had shown that HMGB1 could alter the microglia polarization [21]. Co-staining CD16/32^+^/Iba1^+^ and co-staining CD206^+^/Iba1^+^ were used to mark the M1 and M2 phenotypes of microglia at ischemic penumbra, respectively. Immunofluorescence results showed that the Iba1 was highly expressed in all groups at the edge of injury, suggesting that microglia proliferated at ischemic penumbra. The M2 phenotype marker CD206 increased in the DGA group (Fig. 6C) while the M1 phenotype marker CD16/32 decreased 7 days after DGA treatment (Fig. 6D).

**Fig. 7:**
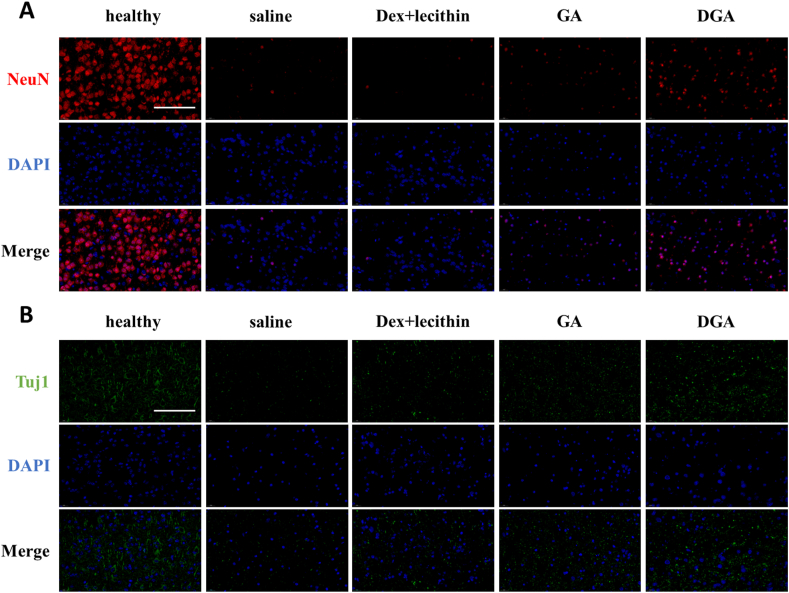
**DGA improved neurogenesis *in vivo*.** (A) Immunofluorescence of NeuN (characteristic nucleoproteins of mature neurons, red) and (B) Tuj1 (tubulin of immature neurons, green) at center of injury site. Nuclei were stained blue; scale bar = 100 μm.

To further verify whether the designed nanoparticles were beneficial for neuron protection and regeneration, immunofluorescence of the mature neuron marker NeuN or immature neuron marker Tuj1 used to visualize the density of neurons. At penumbra areas of brain sections, there were few NeuN^+^ or Tuj1^+^ cells (Figs. S10D and E). This result was consistent with the phenomenon in Fig. 6C and D, the proliferating microglia were dominant at the edge of injury. This phenomenon has been reported in the literature [73]. We further observed the survival of neurons at the center of injury. As shown in Fig. 7A, saline group had fewer mature NeuN^+^ neurons in the central region of injury after 7 days, indicating the injured nerve cells could not spontaneously mature. In contrast, the neurons in the DGA group had gradually matured. In addition, the DGA-treated group also had more Tuj1 markers of newborn neurons (Fig. 7B), suggesting that neurogenesis was still going on. All these results had confirmed that DGA could effectively inhibit the loss of neurons and promote nerve regeneration.

### 3.8 *In vivo* safety evaluation

**Fig. 8:**
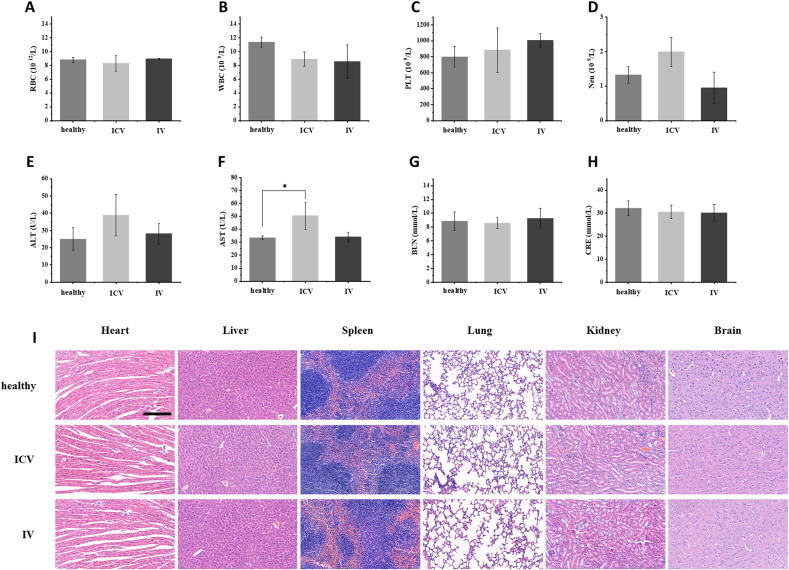
**Safety evaluation *in vivo*.** (A) Routine blood indexes of red blood cell, (B) white blood cells, (C) platelets and (D) neutrophil at day 1 post-injection. (E) Blood biochemical values of alanine aminotransferase, (F) aspartate aminotransferase, (G) blood urea nitrogen, and (H) creatinine at day 1 post-injection (n = 4). (I) H&E staining of heart, liver, spleen, lung, kidney, and brain at day 1 post-injection; scar bar = 200 μm *P < 0.05.

The biosafety of DGA was assessed after treatment through intracerebroventricular injection (ICV) and intravenous injection (IV). Routine blood examination showed the normal levels of RBC, WBC, PLT, and Neu at day 1 and day 7 post-injection (Fig. 8A–D and Figs. S11A–D). The blood biochemical values of ALT, BUN, and CRE did not vary between different treatments as well. AST was a little bit higher in ICV group on day 1, but it was normal at 7 days post-injection, suggesting the liver and kidney functions were not impaired (Fig. 8E–H and Figs. S11E–H). Moreover, there was no significant weight loss with different injection (Fig. S11I) and no distinguishable difference was observed on hematoxylin-eosin (H&E) staining images of heart, liver, spleen, lung, kidney, and brain at day 1 post-injection (Fig. 8I), suggesting no significant toxicity to main organs. Those results supported that safety of DGA nanoparticles and the potential as a safe candidate for brain disease therapy.

## 4 Conclusions

In summary, we prepared ROS-responsive GA-conjugated DEAE-dextran nanoparticles (DGA) and demonstrated their neuroprotective ability by inhibiting HMGB1 and modulating microglia phenotypes to improve the therapeutic effect of stroke. The GA conjugated on polymer exhibited sensitive ROS-induced release manner. In addition, the released GA could effectively exert its inhibitory effect on HMGB1 in cells at low concentrations *in vitro* by virtue of the hydrophilicity polymer. Consistently, *in vivo* tests demonstrated the therapeutic effect on stroke mice, evidenced by smaller infarct volume, better motor function and more neurogenesis. This study provides a simple and effective strategy to regulate microglia, which has potential applications in inhibiting CNS inflammation and promoting nerve regeneration.

## CRediT authorship contribution statement

**Lulu Jin:** Conceptualization, Methodology, Validation, Formal analysis, Investigation, Writing – original draft. **Zhixin Zhu:** Methodology, Validation, Investigation. **Liangjie Hong:** Methodology, Validation, Formal analysis. **Zhefeng Qian:** Methodology, Validation, Formal analysis, Fang Yang: Methodology. **Zhengwei Mao:** Conceptualization, Methodology, Supervision, Writing – review & editing, Funding acquisition.

## Declaration of competing interest

The authors declare that no competing interest exists.
