# Supplementary material for: ROS-responsive 18β-glycyrrhetic acid-conjugated polymeric nanoparticles mediate neuroprotection in ischemic stroke through HMGB1 inhibition and microglia polarization regulation
Source: Bioact Mater. 2022 Apr 1;19:38–49. doi: 10.1016/j.bioactmat.2022.03.040 (PMC8980441; doi:10.1016/j.bioactmat.2022.03.040)
Supplement: Multimedia component 1 [file mmc1.docx]

**Supporting Information**

**ROS-responsive 18β-glycyrrhetic acid-conjugated polymeric nanoparticles mediate neuroprotection in ischemic stroke through HMGB1 inhibition and** **microglia polarization regulation**

Lulu Jin^1^, Zhixin Zhu^1^, Liangjie Hong^1^, Zhefeng Qian^1^, Fang Wang^2^, Zhengwei Mao^1^*

^1^ MOE Key Laboratory of Macromolecular Synthesis and Functionalization, Department of Polymer Science and Engineering, Zhejiang University, Hangzhou 310027, People’s Republic of China;

^2^ The MOE Frontier Science Center for Brain Research and Brain-Machine Integration, Zhejiang University School of Brain Science and Brain Medicine, Hangzhou 310058, China.

* Corresponding authors:

zwmao@zju.edu.cn (Z. M.)

**Synthesis and characterization of NR loaded liposomes (NR@Lip)**

Briefly, lecithin, cholesterol, DSPE-PEG in the molar ratio of 2 : 1 : 0.4 were dissolved in trichloromethane to get 10 mg/mL mixture. Next, 25 μL 1 mg/mL NR dye were added to 0.5 mL mixture in a 50 mL round-bottom flask and the solvent were evaporated slowly by rotary evaporator. Finally, PBS (10 mM) were added to flask and the solution were fully ultrasonic under ice-water for 20 min. All operations containing NR were carried out under dark conditions. In order to confirm that NR was completely loaded in liposome, PBS, artificial cerebral spinal fluid (aCSF), or PBS containing 10% FBS were used as the solvent to obtain 1 mg/mL NR@Lip. After 4 h in a 37°C shaker, Millipore ultrafiltration tubes (MWCO = 3 kDa) were used to obtain the supernatant which was then extracted by DCM. The concentration of NR in the supernatant was measured by fluorescence spectrometer (n = 3). At the same time, the standard curve of NR was also examined.

**Hematoxylin and eosin (H&E) staining**

The collected brains (n = 3 for each group) were fixed in 4% paraformaldehyde for 24 h, dehydrated in graded ethanol, embedded in paraffin, cut into 5 μm-thick sections, and then deparaffinized in xylene, rehydrated through decreasing concentrations of ethanol, washed in PBS. Samples were stained in sequence by hematoxylin for 5 min and eosin for 1 min. After staining, sections were dehydrated through increasing concentrations of ethanol and xylene. Slides were imaged by slide scanner system (Axio Scan. Z1, Zeiss, Germany).

**Immunohistochemistry of HMGB1**

Briefly, antigens were unmasked by microwaving sections in 10 mmol/L citrate buffer, pH = 6.0 (15 min). BSA was used for blocking sections for 20 min at room temperature. Samples were incubated in primary polyclonal rabbit anti-HMGB1 overnight at 4°C, followed by appropriate biotin-conjugated secondary antibody for 30 min at 37°C and subsequently with streptavidin solution for 30 min. The acquired sections were colored with 3,3′-diaminobenzidine and counterstained with hematoxylin. After staining, sections were dehydrated through increasing concentrations of ethanol and xylene. Slides were imaged by slide scanner system (Axio Scan. Z1, Zeiss, Germany) and analyzed by CaseViewer.

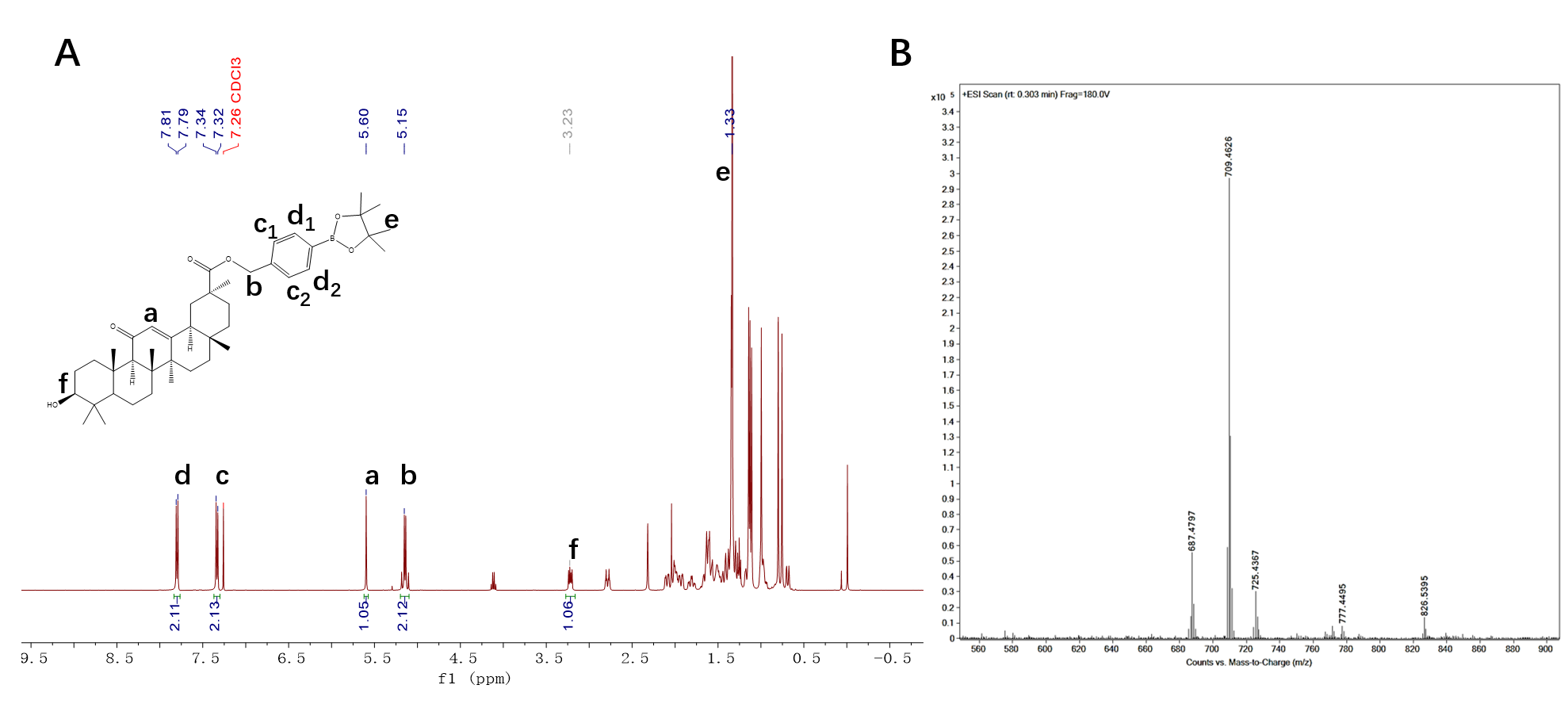


**Figure S1. The chemical structure of compound 1.** (A) ^1^H NMR spectra and (B) MS of the synthesized compound 1 (yield: 72%).

^1^H NMR (400 MHz, Chloroform-*d*) δ 7.80 (d, *J* = 8.0 Hz, 2H), 7.33 (d, *J* = 8.0 Hz, 2H), 5.60 (s, 1H), 5.15 (s, 2H), 3.23 (s, 1H). MS (ESI) m/z: [M + Na]^+^ calculated for C_43_H_63_BO_6_Na, 709.4610; found 709.4626.


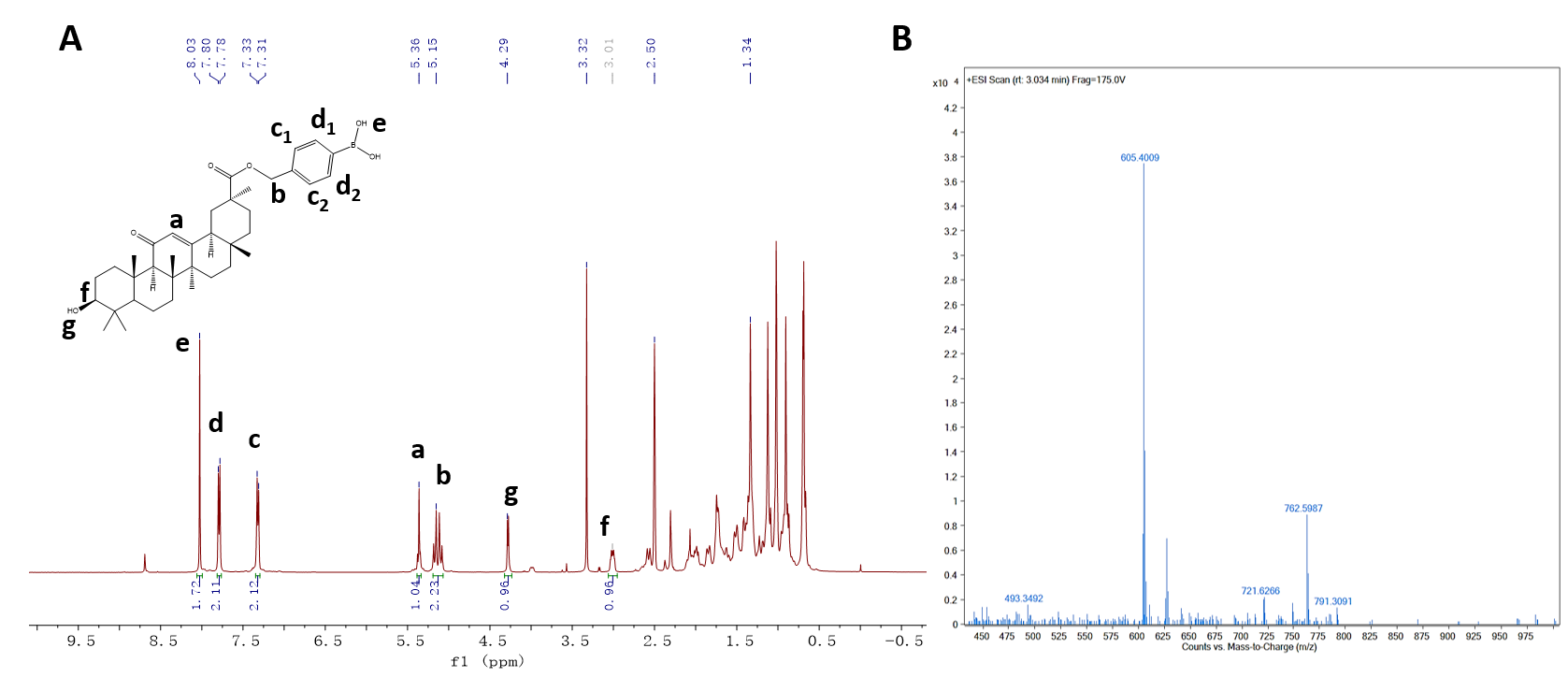


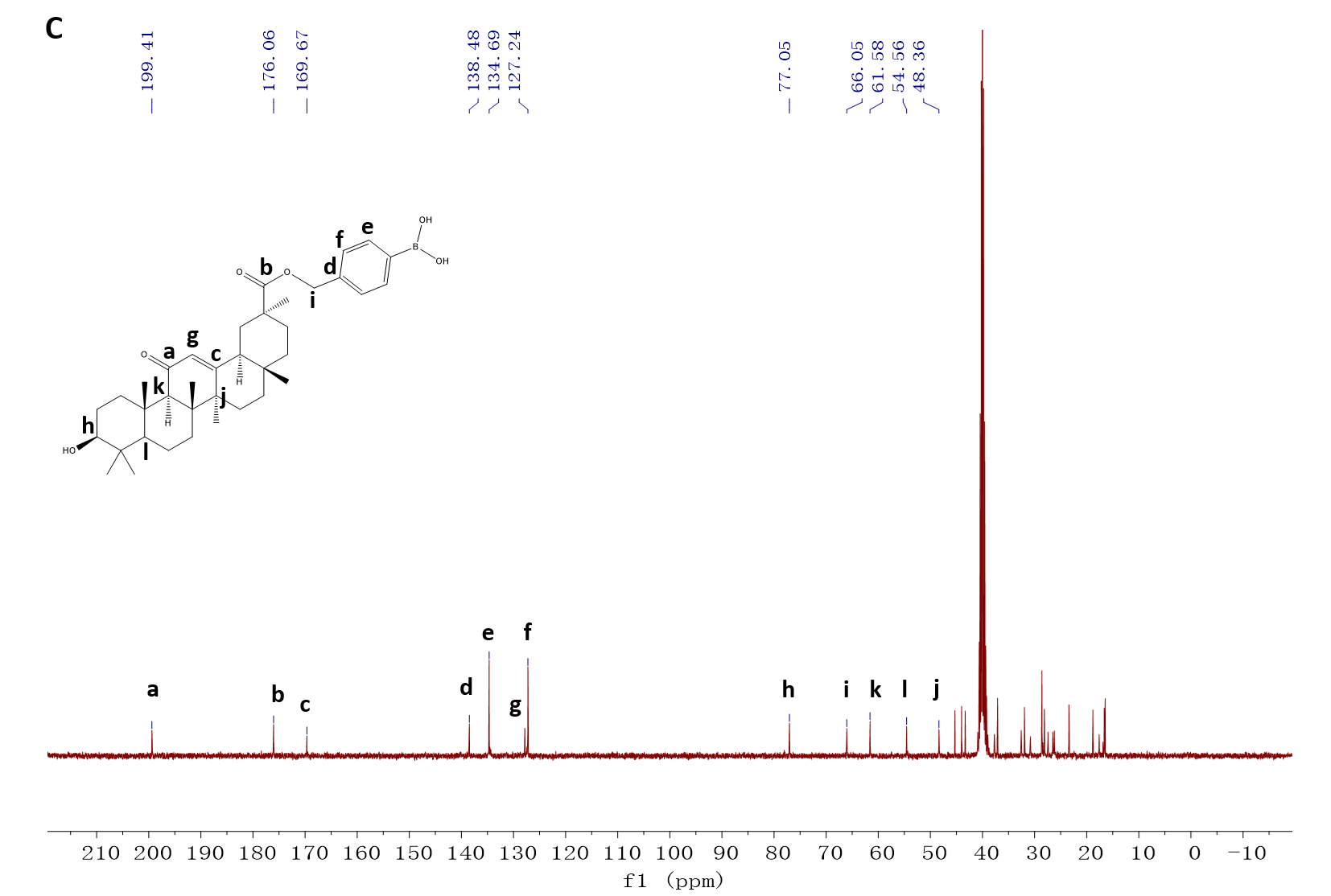


**Figure S2. The chemical structure of compound 2.** (A) ^1^H NMR spectra, (B) MS, and (C) ^13^C NMR spectra of the synthesized compound 2 (yield: 78%).

^1^H NMR (400 MHz, DMSO-*d*_6_): δ 8.03 (s, 2H), 7.79 (d, *J* = 7.8 Hz, 2H), 7.32 (d, *J* = 7.8 Hz, 2H), 5.36 (s, 1H), 5.15 (s, 2H), 4.29 (s, 1H), 3.01 (s, 1H). MS (ESI) m/z: [M + H] ^+^ calculated for C_37_H_53_BO_6_H, 605.4008; found 605.4009. ^13^C NMR (100 MHz, DMSO-*d*_6_): δ 199.41, 176.06, 169.67, 138.48, 134.69, 127.84, 127.24, 77.05, 66.05, 61.58, 54.56, 48.36.


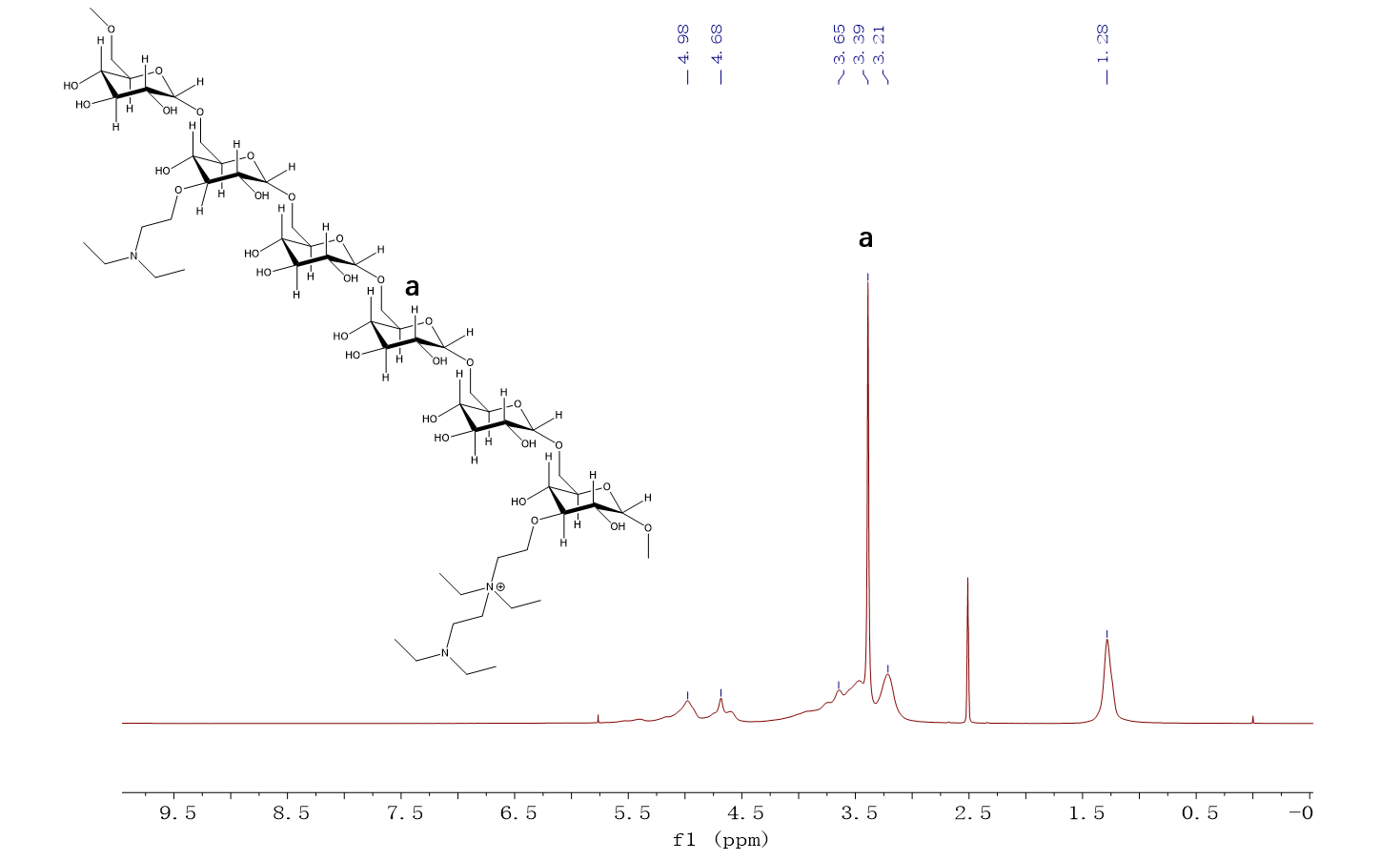


**Figure S3.** The ^1^H NMR (400 MHz, DMSO-*d_6_*) spectra of DEAE-Dex.


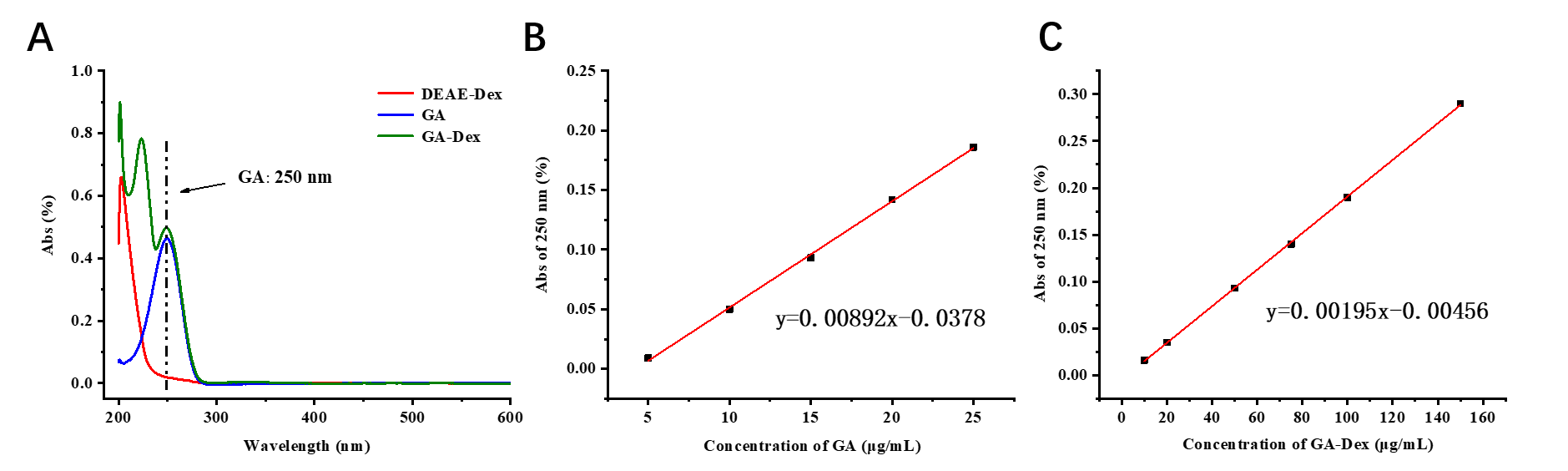


**Figure S4. The GA conjugation ratio.** (A) UV spectra of GA, DEAE-Dex, and GA-Dex in methanol. (B) GA and (C) GA-Dex standard curve at 250 nm.

GA standard curve at 250 nm: y_GA_ = 0.00892·x_GA_ - 0.0378

GA-Dex standard curve at 250 nm: y_GA-Dex_ = 0.00195·x_GA-Dex_ - 0.00456

Suppose x_GA_ / x_GA-Dex_ = k (fixed value), the influence of GA-Dex at 224 nm on 250 nm = a (fixed value).

Thus, y_GA-Dex_ - y_GA_ = a = (0.00195 - 0.00892·k)·x_GA-Dex_ + 0.03324

Because a and k are fixed. So, a = 0.003324, k = 0.00195/0.00892 = 0.2186

Drug loading efficiency was 21.86%


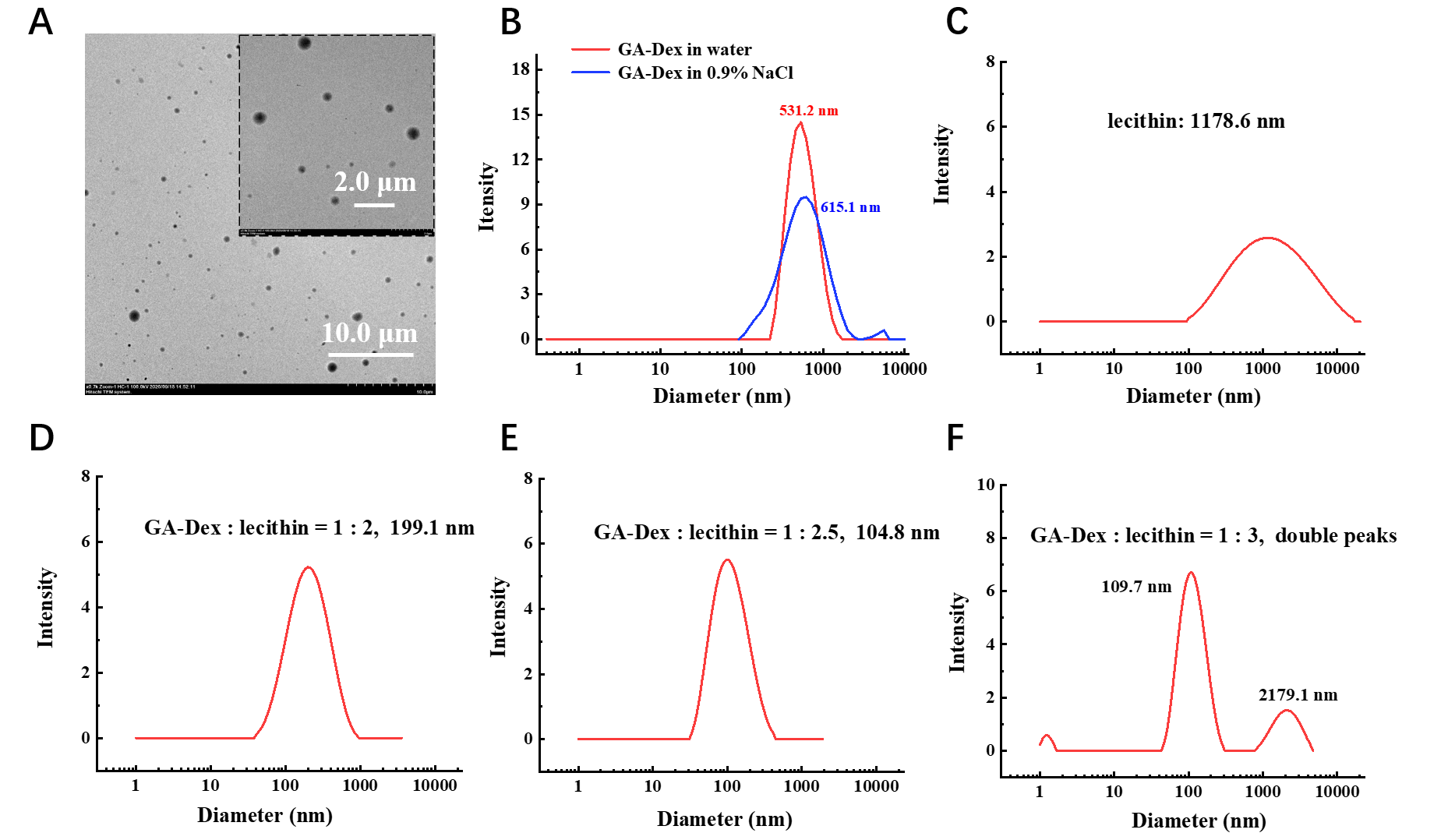


**Figure S5. Characterization of GA-Dex and DGA.** (A) TEM images of GA-Dex freeze-dried in water. (B) Average hydrodynamic diameters of GA-Dex in water and 0.9% NaCl. (C-F) Average hydrodynamic diameters of lecithin (C), GA-Dex : lecithin = 1 : 2 (D), GA-Dex : lecithin = 1 : 2.5 (E), GA-Dex : lecithin = 1 : 3 (F) in water.


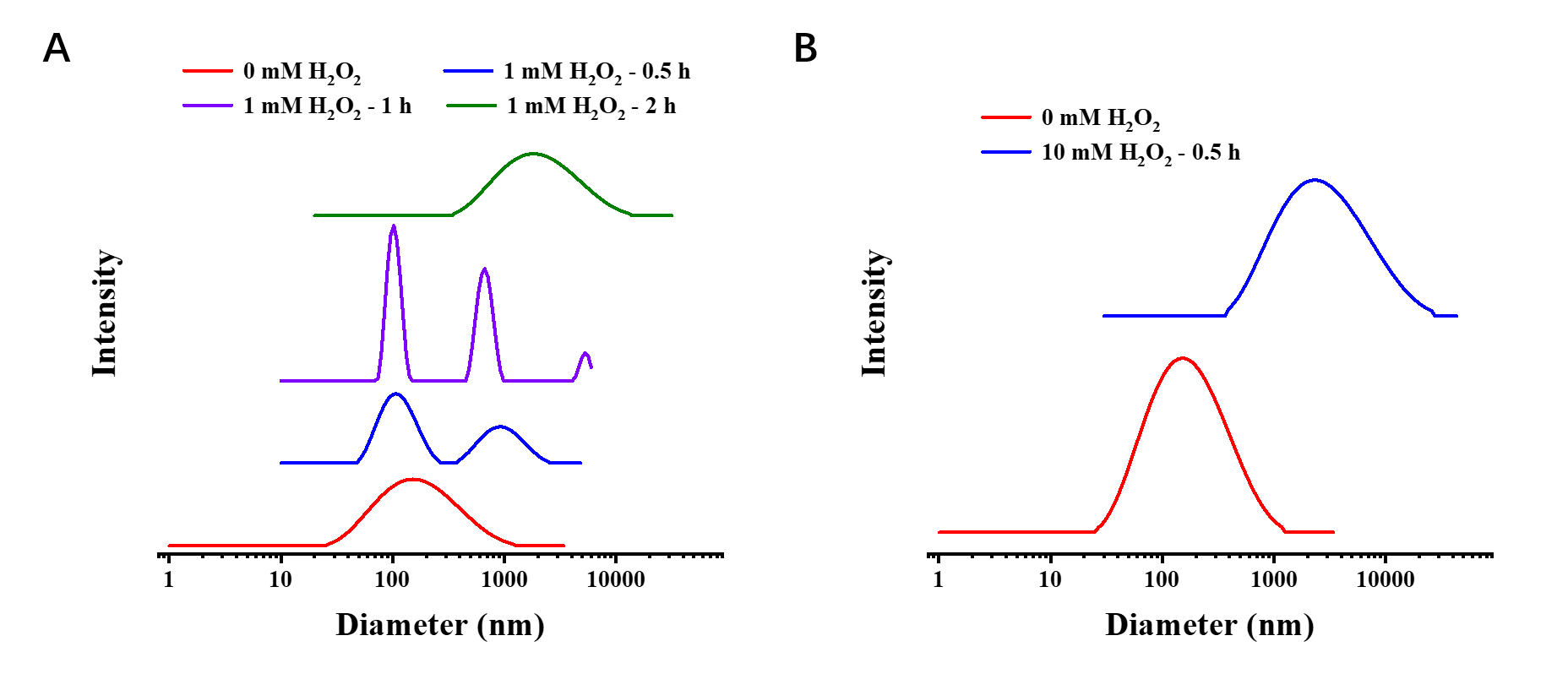


**Figure S6. H_2_O_2_-responsiveness of DGA.** (A) DGA in saline with 1 mM H_2_O_2_ or (B) 10 mM H_2_O_2_ at 37°C.


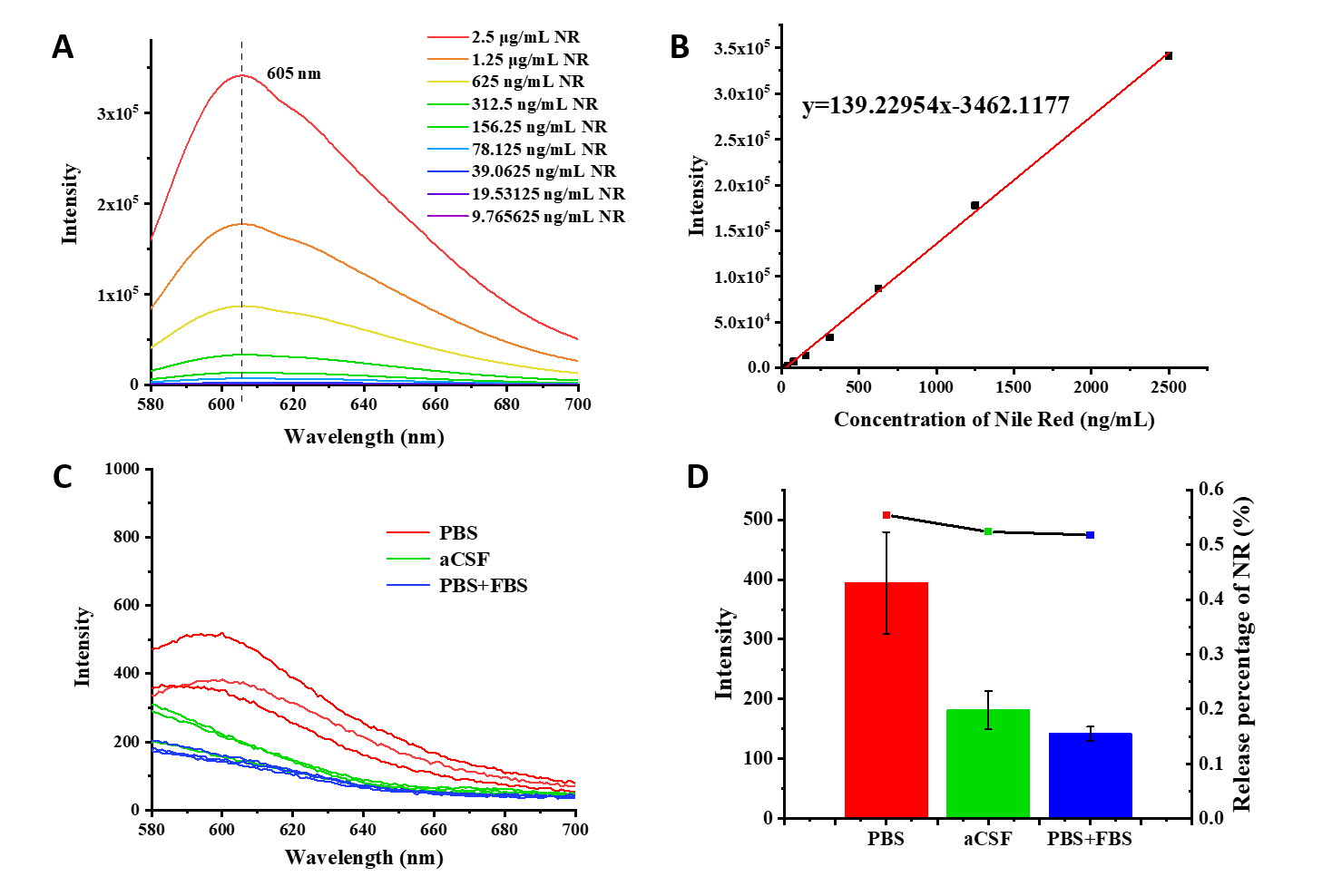


**Figure S7. Stability of NR dye in NR@Lip.** (A) Fluorescence spectrum of a series concentrations of NR. (B) Standard curve of NR at 605 nm. (C) The fluorescence spectrum of supernatant after 1 mg/mL NR@Lip were dispersed into PBS, aCSF, and PBS containing 10%FBS and incubated for 4 h. (D) Average fluorescence intensity and the percentage of NR released in different conditions.


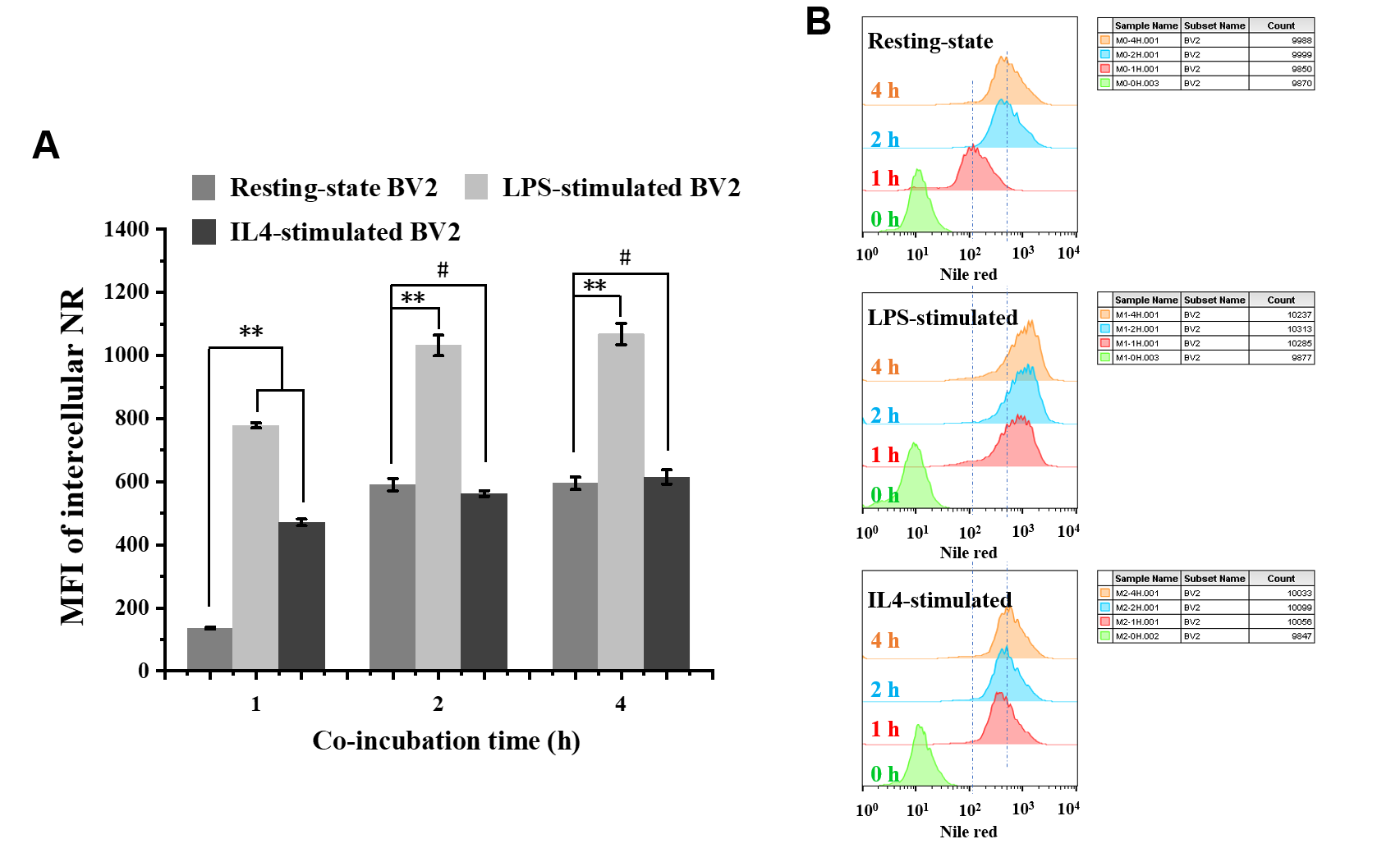


**Figure S8. The phagocytosis of BV2 cells.** (A) Quantified analysis of NR@Lip in BV2 cells with flow cytometry by increasing of co-incubation time (n = 3). The mean fluorescence intensity (MFI) was relative to the cells without NR@Lip addition. (B) Typical flow cytometry data of cellular uptake of NR@Lip in different treated BV2 cells. Data are presented as mean ± SD. **p < 0.01, # represented no significant difference.


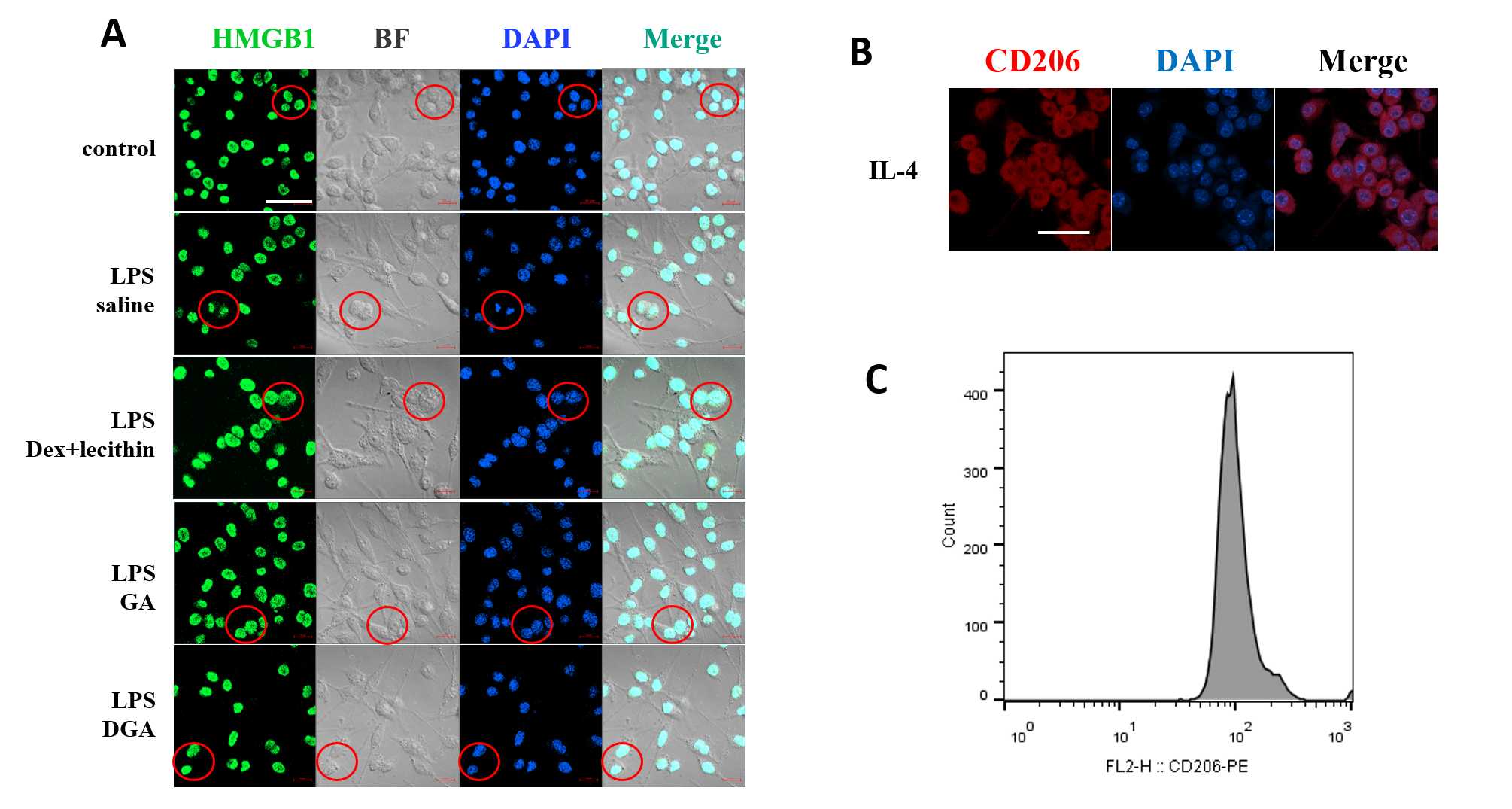


**Figure S9. Inhibition of HMGB1 translocation and transformation of microglia phenotype *in vitro*.** (A) Cellular immunofluorescence images of HMGB1 after 1 μg/mL LPS stimulation for 12 h, followed by saline, Dex+lecithin, GA or DGA treatment for 12 h. Untreated resting-state BV2 cells were set as control. HMGB1 was stained green and nuclei were stained blue. The gray images were bright field images from which cell outlines could be observed; scar bar = 50 µm. (B) Represented immunofluorescence staining images of M2 phenotype marker CD206 (red) in BV2 cells after IL-4 treatment. The gray images were bright field images from which cell outlines could be observed. Nuclei were counterstained with DAPI (blue); scar bar = 50 µm. (C) Typical flow cytometry data of CD206 after IL-4 treatment.


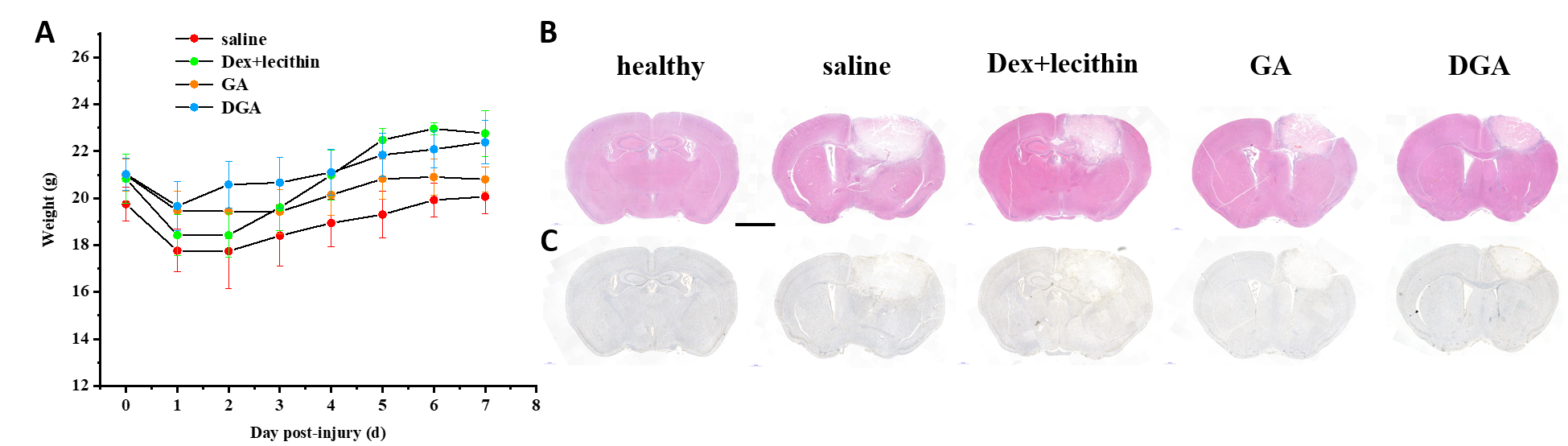

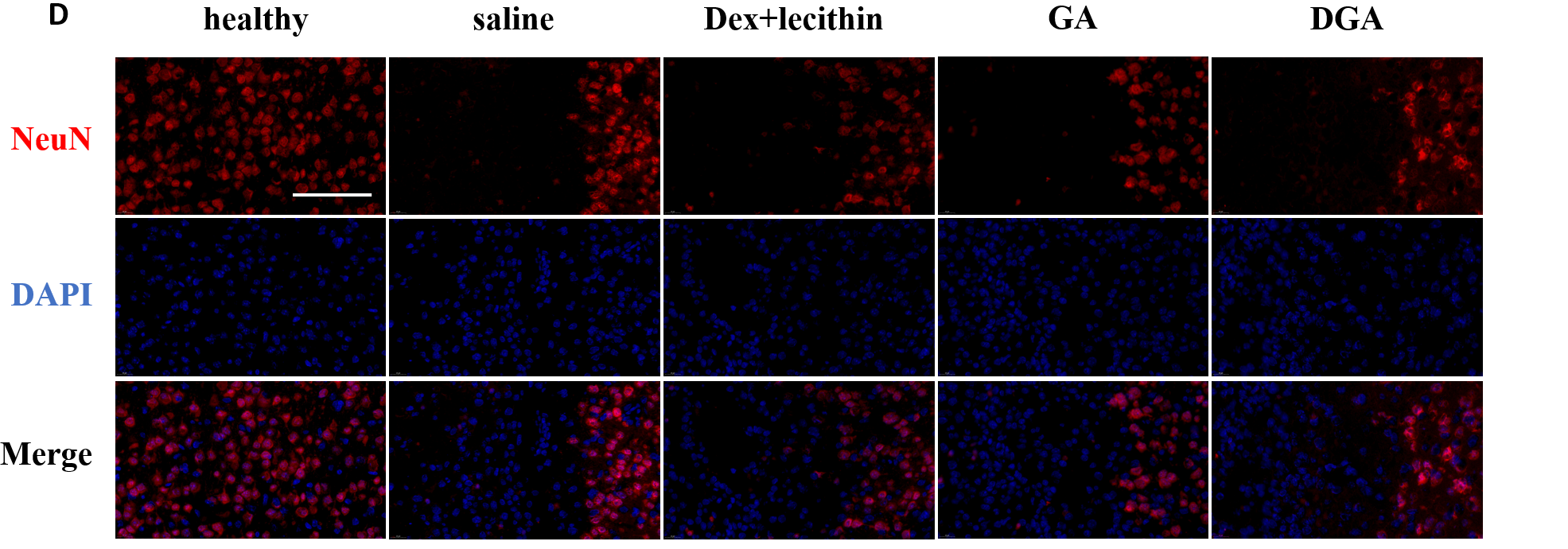

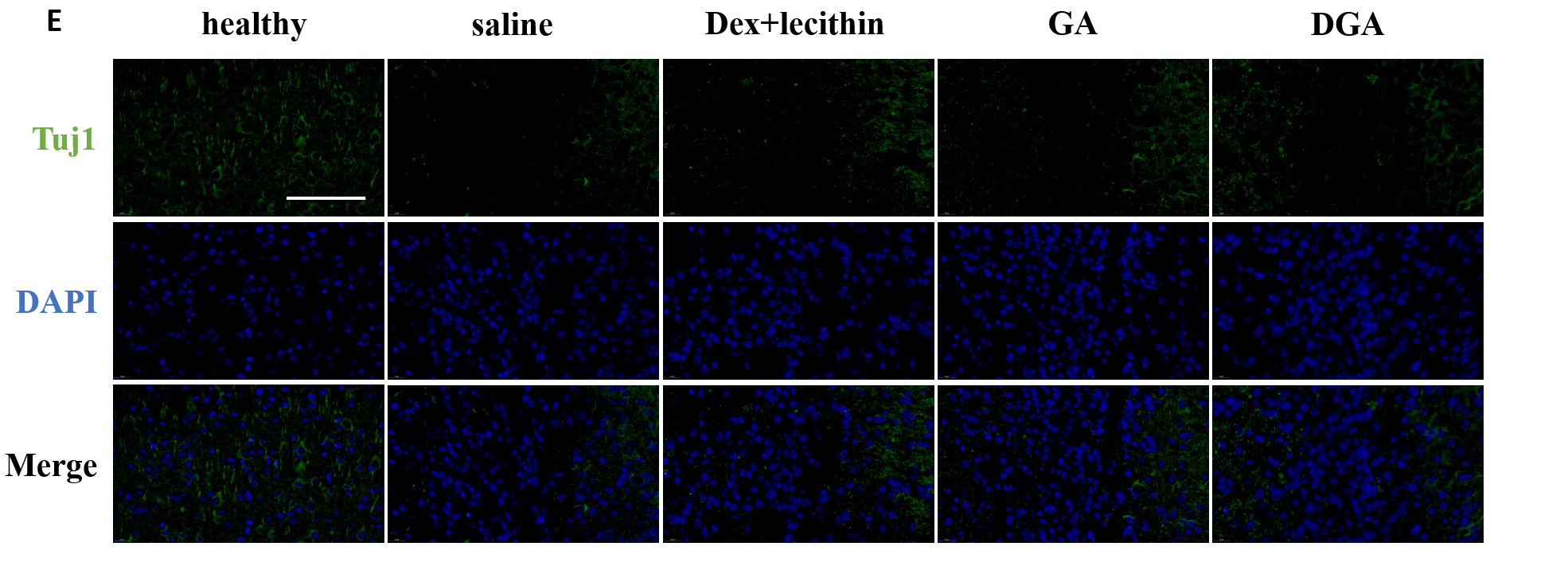


**Figure S10. The therapeutic effect of DGA after stroke *in vivo*.** (A) Body weight before cerebral infarction (day 0) and after stroke with different treatments (n = 5). (B) H&E staining and (C) immunohistochemistry of HMGB1 in representative brain sections; scar bar = 2 mm. (D) Immunofluorescence of NeuN (characteristic nucleoproteins of mature neurons, red) and (E) Tuj1 (tubulin of immature neurons, green) at periphery of injury site. Nuclei were stained blue; scale bar = 100 µm. On the left, center, and right of each image were lesion, boundary, and normal tissue.


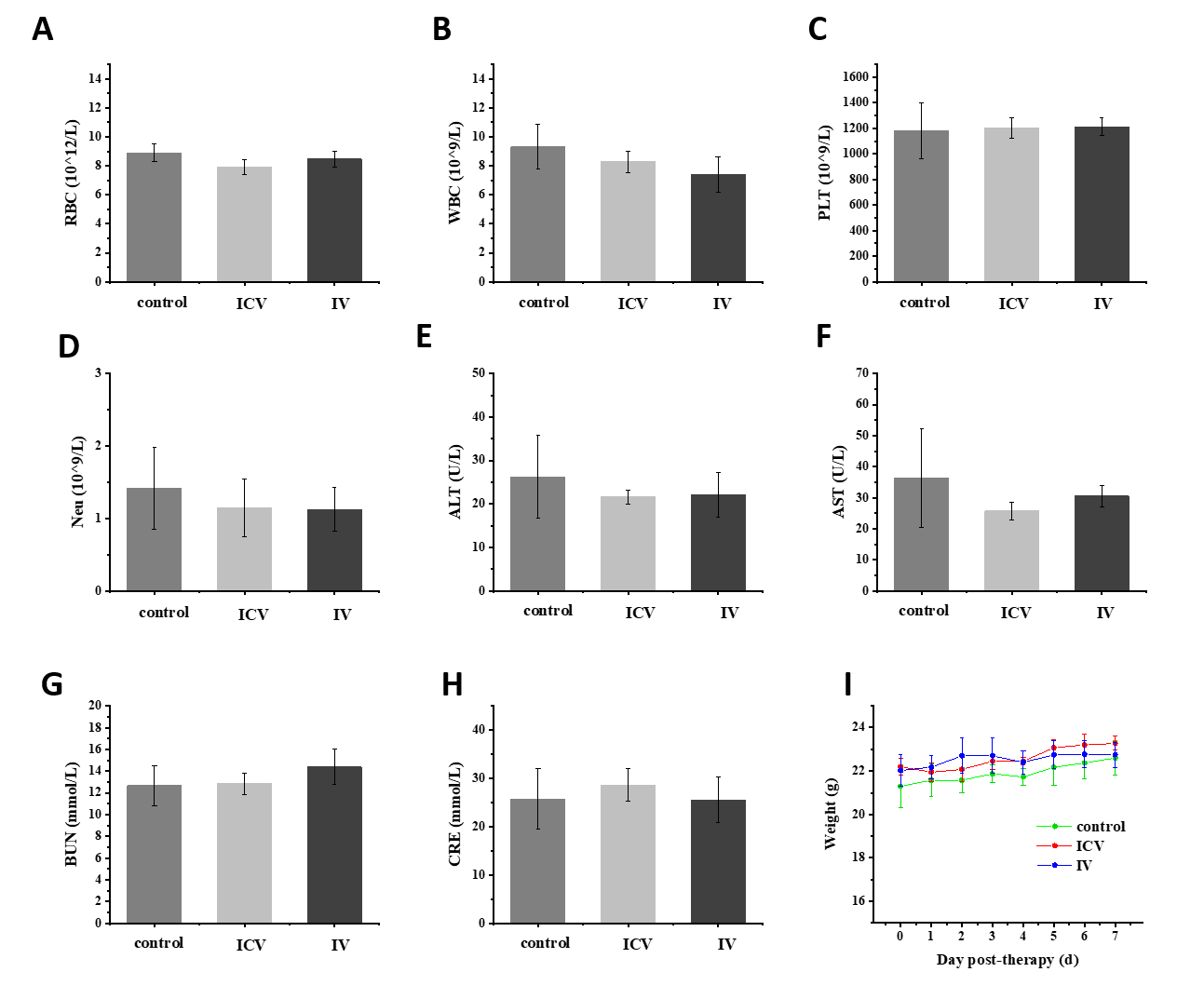


**Figure S11. Toxicological evaluations after 7 days of treatment via different injections.** (A-D) Routine blood indexes of red blood cell (A), white blood cells (B), platelets (C) and neutrophil (D) at day 7 post-injection. (E-H) Blood biochemical values of alanine aminotransferase (E), aspartate aminotransferase (F), blood urea nitrogen (G), and creatinine (H) at day 7 post-injection (n = 4). (I) Body weight before therapy (day 0) and after intracerebroventricular injection and intravenous injection within 7 days (n = 4).

.
